# Museomics Unveil the Phylogeny and Biogeography of the Neglected Juan Fernandez Archipelago *Megalachne* and *Podophorus* Endemic Grasses and Their Connection With Relict Pampean-Ventanian Fescues

**DOI:** 10.3389/fpls.2020.00819

**Published:** 2020-06-26

**Authors:** María Fernanda Moreno-Aguilar, Itziar Arnelas, Aminael Sánchez-Rodríguez, Juan Viruel, Pilar Catalán

**Affiliations:** ^1^Escuela Politécnica Superior de Huesca, Universidad de Zaragoza, Huesca, Spain; ^2^Departamento de Ciencias Biológicas, Universidad Técnica Particular de Loja, Loja, Ecuador; ^3^Royal Botanic Gardens, Kew, Richmond, United Kingdom; ^4^Grupo de Bioquímica, Biofísica y Biología Computacional (BIFI, UNIZAR), Unidad Asociada al CSIC, Zaragoza, Spain; ^5^Department of Botany, Institute of Biology, Tomsk State University, Tomsk, Russia

**Keywords:** ancestral range reconstruction, endemic Loliinae grasses, Fernandezian clade, genome skimming, phylogenomics, taxonomically neglected species

## Abstract

Oceanic islands constitute natural laboratories to study plant speciation and biogeographic patterns of island endemics. Juan Fernandez is a southern Pacific archipelago consisting of three small oceanic islands located 600–700 km west of the Chilean coastline. Exposed to current cold seasonal oceanic climate, these 5.8–1 Ma old islands harbor a remarkable endemic flora. All known Fernandezian endemic grass species belong to two genera, *Megalachne* and *Podophorus*, of uncertain taxonomic adscription. Classical and modern classifications have placed them either in Bromeae (*Bromus*), Duthieinae, Aveneae/Poeae, or Loliinae (fine-leaved *Festuca*); however, none of them have clarified their evolutionary relationships with respect to their closest *Festuca* relatives. *Megalachne* includes four species, which are endemic to Masatierra (Robinson Crusoe island) (*M. berteroniana* and *M. robinsoniana*) and to Masafuera (Alejandro Selkirk island) (*M. masafuerana* and *M. dantonii*). The monotypic *Podophorus bromoides* is a rare endemic species to Masatierra which is only known from its type locality and is currently considered extinct. We have used museomic approaches to uncover the challenging evolutionary history of these endemic grasses and to infer the divergence and dispersal patterns from their ancestors. Genome skimming data were produced from herbarium samples of *M. berteroniana* and *M. masafuerana*, and the 164 years old type specimen of *P. bromoides*, as well as for a collection of 33 species representing the main broad- and fine-leaved Loliinae lineages. Paired-end reads were successfully mapped to plastomes and nuclear ribosomal cistrons of reference *Festuca* species and used to reconstruct phylogenetic trees. Filtered ITS and *trn*TLF sequences from these genomes were further combined with our large Loliinae data sets for accurate biogeographic reconstruction. Nuclear and plastome data recovered a strongly supported fine-leaved Fernandezian clade where *Podophorus* was resolved as sister to *Megalachne*. Bayesian divergence dating and dispersal-extinction-cladogenesis range evolution analyses estimated the split of the Fernandezian clade from its ancestral southern American Pampas-Ventanian Loliinae lineage in the Miocene-Pliocene transition, following a long distance dispersal from the continent to the uplifted volcanic palaeo-island of Santa Clara-Masatierra. Consecutive Pliocene-Pleistocene splits and a Masatierra-to-Masafuera dispersal paved the way for *in situ* speciation of *Podophorus* and *Megalachne* taxa.

## Introduction

Genomic data are increasingly called upon to elucidate evolutionary and taxonomic challenges posed by several cryptic or ambiguously related organisms, which could not be resolved using traditional approaches, such as morphometrics or standard molecular methods (Harrison and Kidner, 2011; Straub et al., 2012; Carter et al., 2020; Larridon et al., 2020). The advent of the high-throughput sequencing (HTS) methods have outpaced classical molecular barcoding and phylogenetic procedures based on few molecular markers that have served to build phylogenies with constrained resolution limits (Diaz-Perez et al., 2018; Sancho et al., 2018). While the results obtained from the genomic-based approaches are overall congruent with previous findings based on reduced sets of genes and genetic markers (Saarela et al., 2018), the thoroughly dissection of genomes have untapped large sets of taxonomically informative gene copy variants or single nucleotide polymorphism (SNPs) and have allowed the reconstruction of better resolved and more strongly supported phylogenies (Soltis et al., 2018). These new metadata have facilitated the identification of previously neglected cryptic taxa (Spriggs et al., 2019) and the construction of more robust phylogenetic trees where the evolutionary positions of previously unknown, doubtful, or ambiguous lineages have been elucidated in some cases (Leebens-Mack et al., 2019; Li et al., 2019).

The application of HTS methods to the analysis of museum collections, defined as museomics, has revolutionized the study of the organismic diversity (Besnard et al., 2014; Nevill et al., 2020). Plant herbarium specimens were occasionally used in traditional phylogenetic and population genetic studies due to the poor preservation of the specimens or their low quality DNA. Herbarium specimens have been progressively incorporated to taxonomic and evolutionary studies using HTS methods thanks to the simultaneous generation of a large quantity of sequences for the different genomes present in an organism (Straub et al., 2012; Besnard et al., 2014). Among the HTS approaches used with both herbarium and fresh collections, genome skimming (Dodsworth, 2015; Richter et al., 2015) has been successfully applied to reconstruct DNA genomes and regions that exist in multiple copies, such as plastomes, mitomes and the nuclear ribosomal cistron, and even some nuclear single copy genes (Besnard et al., 2014). Among other advances, museomics has untapped the placement of recently extinct taxa in phylogenies (Sebastian et al., 2010; Welch et al., 2016; Zedane et al., 2016; Silva et al., 2017). Thus, the combined use of current and extinct plant species samples, and of herbarium and recently collected samples allows to uncover largely sampled phylogenetic trees of plant lineages (Malakasi et al., 2019).

**FIGURE 1:**
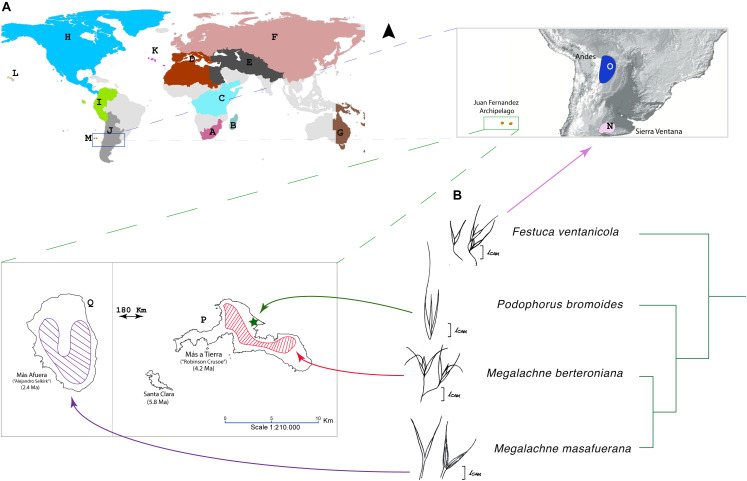
**(A)** Distribution map of the Juan Fernandez archipelago *Megalachne* and *Podophorus* species included in this study [*M. berteroniana* and *M. masafuerana* distributions have been obtained from Peña et al. (2017) and Penneckamp-Furniel and Villegas (2019); *P. bromoides* is tentatively mapped in the Cumberley bay of Masatierra; cf. Baeza et al. (2007)] and of the Pampean-Ventanian *Festuca ventanicola*, showing the world and the American-Vulpia-Pampas Operational areas (OAs) used in the biogeographical analyses of Loliinae. OAs: Loliinae DEC model: A – South Africa; B – Madagascar and Mascarenes; C – Tropical Africa; D – Mediterranean; E – Irano-Turanian-Himalayan region; F – Eurasia (Eurosiberian region); G – Australasia (New Zealand, south-western Australia, Papua-New Guinea); H – North and Central America; I – Northern Andes; J – Southern Andes and Southern South America; K – Macaronesia; L – Hawaii; M – Juan Fernández; American-Vulpia-Pampas DEC model: H – North and Central America; N – Pampas-Ventania; O – Andes; P – Masatierra; Q – Masafuera. **(B)** Phylogenetic ITS-TLF subtree showing the relationships of the Fernandezian *Podophorus bromoides*, *Megalachne berteroniana*, and *M. masafuerana* grasses and its close relative *Festuca ventanicola*; drawns of the floral “vulpioid” phenotype are shown for each species.

Oceanic archipelagos have been recognized as hotspots of diversity and natural laboratories for long distance colonization and plant speciation events (Triantis et al., 2016). Juan Fernandez is one of the smallest oceanic archipelagos. It consists of three small islands located in the southern Pacific, 580–730 km offshore of the western Chilean coast [Masatierra or Robinson Crusoe (47.94 km^2^, 0–915 masl), Masafuera or Alejandro Selkirk (49.52 km^2^, 0–1,319 masl), and Santa Clara (2.21 km^2^, 0–350 masl)] (Stuessy et al., 1992, 2017). The two main islands have similar sizes but differ in plant communities and diverse grassland extensions due to their different ages and erosional patterns (Greimler et al., 2017), and are separated each other by 181 km (Figure 1). The current Fernandezian volcanic islands are relatively young (Stuessy et al., 1984). Despite its total small area (100.2 km^2^), the archipelago harbors one of the richest endemic floras (60% vascular species, 11% genera, 1 paleoherb family; Stuessy et al., 1992). Floristic studies indicate that 55 grass species grow in Juan Fernandez archipelago; most of them are invasive taxa except five endemic species that belong to the Fernandezian *Megalachne* Steud. and *Podophorus* Phil. genera (Baeza et al., 2007; Peña et al., 2017; Penneckamp-Furniel and Villegas, 2019). *Megalachne* and the monotypic genus *Podophorus* have been historically assigned to different temperate grass tribes. *Megalachne* was originally described by Steudel in 1854 as close to *Bromus* (it was also described as *Pathantera* by Philippi in 1856), though they differ in the number and disposition of the stigmas (three apical in *Megalachne*, two subapical in *Bromus*) and the shape of the glume apex (aristulate in *Megalachne*, mutique in *Bromus;* Hackel, 1887). However, Pilger in 1920 and Skottsberg in 1922 transferred, respectively, *Megalachne* and *Pathantera* to *Bromus*, based on the sharing of laterally compressed spikelets and keeled lemmas, such as those in *Bromus* sect. *Ceratochloa* (Peña et al., 2017) thus classifying them within tribe Bromeae. In 1954, Pilger recognized *Megalachne* as a separate genus from *Bromus* (Peña et al., 2017); Tateoka (1962) using evidences from the morphology and apical hairiness of the ovary, apical emergence of stigmas, and type of starch grains and serology, suggested the proximity of *Megalachne* to *Festuca*, thus attributing it to tribe Poeae (subtribe Loliinae). The taxonomic adscription of *Megalachne* and *Podophorus* to tribe Poeae was accepted in most grass classifications though Soreng et al. (2003) assigned them initially to tribe Stipeae subtribe Duthieinae based on the overall habit resemblance. Nonetheless, the comprehensive morphological and molecular study of the newly delimited tribe Duthieeae of Schneider et al. (2011) demonstrated, using ITS sequences, that *Megalachne* and *Podophorus* were not part of this early diverging pooid lineage, and suggested that they likely belonged to the Aveneae/Poeae complex. In recent studies, phylogenetic analyses conducted by Schneider et al. (2012) and Tkach et al. (2020) using, respectively, nuclear ITS and plastid *mat*K sequences and nuclear ITS and ETS and plastid *mat*K, *trn*K, and *trn*LF sequences corroborated it, showing that *Megalachne* was nested within the fine-leaved Loliinae clade.

*Megalachne* and *Podophorus* differentiate from each other in the number of florets per spikelet [3–6 in *Megalachne*, 1–(+1 sterile) in *Podophorus*), the type of lemma (keeled vs. rounded], the length of the glumes (equal vs. shorter than anthecium) and the prolongation of the rachilla apex (shorther vs. equal than anthecium; Baeza et al., 2007; Kellogg, 2015; Peña et al., 2017). *Megalachne* consisted until recently of two species, *M. berteroniana* Steud. and *M. masafuerana* (Skottsb. & Pilg. ex. Pilg.) Matthei, endemic to the Masatierra and Masafuera islands, respectively (Baeza et al., 2007). Both species grow in coastal and mountain cliffs in their respective islands (Danton et al., 2006). Recent morphological studies have identified two new species, *M. robinsoniana* C. Peña, endemic to Masatierra (Peña et al., 2017), and *M. dantonii* Penneck. & Gl. Rojas, endemic to Masafuera (Penneckamp-Furniel and Villegas, 2019). The four *Megalachne* species differ in the lengths of the lemma and glume awns and the number of florets per spikelet (Peña et al., 2017; Penneckamp-Furniel and Villegas, 2019). The systematic and evolutionary fate of *Podophorus* is more enigmatic. Its single species *P. bromoides* Phil. is only known from its type specimens, collected in Masatierra and described by Philippi in 1856, and is currently considered to be extinct (Baeza et al., 2007).

Loliinae is one of the largest subtribes of the temperate pooid grasses and contains pasture and forage species of high ecological and economic importance. Its largest genus *Festuca* is formed by ∼600 worldwide distributed species inhabiting cool seasonal regions of both hemispheres and high tropical mountains (Catalan, 2006). Molecular phylogenetic studies have shown that *Festuca* is largely paraphyletic (Catalan et al., 2007; Inda et al., 2008; Minaya et al., 2017). Recent studies, based on the Schneider et al. (2011, 2012) ITS and *mat*K data and previous morphological findings, reclassified *Megalachne* and *Podophorus* within subtribe Loliinae (Soreng et al., 2015, 2017), however, they did not identify the closest relatives of these Fernandezian grasses. The phylogenetic relationships obtained by previous authors for the three studied *Megalachne* and *Podophorus* taxa were also taxonomically incongruent, showing a closer relationship of *M. berteroniana* to *P. bromoides* than to its congener *M. masafuerana* (Schneider et al., 2011).

Here we have used a museomic approach based on genome skimming data to uncover the phylogenetic and biogeographical history of the neglected Fernandezian *Megalachne* and *Podophorus* grasses. The aims of our study were to (i) infer the phylogeny of *Megalachne* and *Podophorus* within a large sample representation of Loliinae lineages; (ii) identify the closest relatives of the Fernandezian grasses; (iii) reconstruct the relationships among the *Megalachne* and *Podophorus* taxa; (iv) estimate divergence times of the Fernandezian lineages; and (v) infer the colonization patterns and speciation events of the ancestors of *Megalachne* and *Podophorus* in the Juan Fernandez islands.

## Materials and Methods

### Sampling

Representative samples of *Megalachne*, *Podophorus*, and other Loliinae genera were included in the study (Figure 1 and Table 1). Herbarium samples of *Megalachne berteroniana* and *M. masafuerana* provided by the Oregon State University Herbarium (OSC11751 and OSC9150 collections; Table 1) were used to isolate high quality and quantity DNA for genome sequencing and downstream evolutionary analyses. A herbarium sample of *M. robinsoniana* provided by the Concepción University Herbarium (CONC40598 collection) failed to generate good quality DNA for the study. The recently described *M. dantonii* species (Penneckamp-Furniel and Villegas, 2019) could not be included in our study. A 164 years old sample of the currently considered extinct *Podophorus bromoides* Phil., only known from its three type specimens, was provided by the Royal Botanic Gardens Kew’s Herbarium (Philippi 1861, isotype collection; Table 1)^1^ and was successfully used for genome skimming sequencing and downstream analysis. In our aim to identify the closest relatives of the Fernandezian *Megalachne* and *Podophorus* grasses, DNA was also isolated from 33 Loliinae samples (Table 1) representing all the known broad-leaved, intermediate, and fine-leaved Loliinae lineages (Inda et al., 2008; Minaya et al., 2017) and used for genome skimming sequencing and phylogenomic analyses. Some of these samples were collected from poorly explored geographical areas, including four new *Festuca* samples from South America, the putative region of origin of the ancestors of *Megalachne* and *Podophorus* (Stuessy et al., 2017) and two from Tropical Africa and South Africa. In addition, two new Loliinae samples from South America and one sample from South Africa not studied before (Table 1) were sequenced for the nuclear ITS (ITS1-5.8S-ITS2) and the plastid *trn*TL (*trn*T-*trn*L intergenic spacer) and *trn*LF (*trn*A-Leu, *trn*L-*trn*F intergenic spacer, *trn*A-Phe) loci, together with 97 samples from a wide-sampling of all currently known Loliinae lineages (Inda et al., 2008; Minaya et al., 2017). Although species of *Megalachne* and *Podophorus* and other fine-leaved Loliinae genera have been synonymized to *Festuca*, and those of broad-leaved Loliinae to different festucoid genera in recent studies (Soreng et al., 2017; Tkach et al., 2020), we follow the *Festuca* sensu lato classification of Catalan et al. (2007) which is based on an evolutionary systematic criterion that is nomenclaturally conservative and maintains a paraphyletic *Festuca* (with subgenera and sections) and other traditionally recognized genera until more complete phylogenetic studies of Loliinae are conducted. We have selected this scenario because of present uncertainties about the phylogeny of several Loliinae lineages and taxonomic and nomenclatural instability of the *Festuca* sensu stricto (i.e., fine-leaved Loliinae lineages) classification, that would leave some broad-leaved Loliinae lineages without name or with unclear adscription (e.g., some broad-leaved “*Festuca*”). It could be possible, however, that genera phylogenetically embedded within the large Loliinae clade or its fine-leaved subclade would be subordinated to *Festuca*, once all or most of the Loliinae taxa are phylogenetically analyzed and consistent synapomorphies are defined. At this respect, nomenclatural combinations have been proposed for the fine-leaved *Megalachne* (*Festuca megalachna* Röser & Tkach; *F. masafuerana* (Skottsb. & Pilg. ex Pilg.) Röser & Tkach; *F. robinsoniana* (C.M.Peña) Röser & Tkach; *F. dolichathera* Röser & Tkach) and *Podophorus* (*F. masatierrae* Röser & Tkach) species synonymized to *Festuca* (Tkach et al., 2020). Sixteen additional species were added as outgroups to provide reliable fossil calibration points for molecular dating (Supplementary Table S1).

**TABLE 1:** List of taxa included in the phylogenomic study of the Fernandezian and other Loliinae grasses.

Taxon	Source	Ploidy	No. reads	Genbank/Phytozome accession No.	
				Plastome	rDNA cistron
					
*Festuca abyssinica*	Tanzania: Kilimanjaro	4x	12041	SAMN14647043	MT145276
*Festuca africana*	Uganda: Bwindi forest	10x	13549	SAMN14647044	MT145277
*Festuca amplissima*	Mexico: Barranca del Cobre	6x	12058	SAMN14647045	MT145278
*Festuca arundinacea* var. *letourneuxiana*	Morocco: Atlas Mountains	10x	16839	SAMN14647059	MT145292
*Festuca asplundii*	Ecuador: Saraguro	6x	25088	SAMN14647046	MT145279
*Festuca caldasii*	Ecuador: Las Chinchas –Tambara	?	9863	SAMN14647047	MT145280
*Festuca capillifolia*	España: Cazorla	2x	13430	SAMN14647048	MT145281
*Festuca chimborazensis*	Ecuador: Chimborazo-Cotopaxi	4x	10913	SAMN14647049	MT145282
*Festuca durandoi*	Portugal: Alto do Espinheiro	2x	12688	SAMN14647050	MT145283
*Festuca eskia*	Spain: Picos de Europa	2x	24041	SAMN14647051	MT145284
*Festuca fenas*	Spain: Madrid	4x	16112	SAMN14647052	MT145285
*Festuca fimbriata*	Argentina: Apóstoles	6x	15741	SAMN14647053	MT145286
*Festuca fontqueri*	Morocco: Rif, Outa-El-Kadir	2x	22187	SAMN14647054	MT145287
*Festuca gracillima*	Argentina: Tierra de Fuego	6x	13888	SAMN14647055	MT145288
*Festuca holubii*	Ecuador: Saraguro	?	10264	SAMN14647056	MT145289
*Festuca francoi*	Portugal: Azores	2x	17592	SAMN14647057	MT145290
*Festuca lasto*	Spain: Los Alcornocales	2x	21581	SAMN14647058	MT145291
*Festuca mairei*	Morocco: Atlas Mountains	4x	19134	SAMN14647060	MT145293
*Festuca molokaiensis*	USA: Molokai	?	12188	SAMN14647061	MT145294
*Festuca ovina*	Russia: Gatchinskii Raion	2x	11364	SAMN14647062	MT145295
*Festuca pampeana*	Argentina: Sierra Ventana	6x	14862	SAMN14647063	MT145296
*Festuca paniculata*	Spain: Puerto de los Castaños	2x	35808	SAMN14647064	MT145297
*Festuca parvigluma*	China: Baotianman	4x	15872	SAMN14647065	MT145298
*Festuca pratensis*	England: USDA/283306	2x	30021	SAMN14647066	MT145301
*Festuca procera*	Ecuador: Riobamba	4x	12189	SAMN14647067	MT145299
*Festuca pyrenaica*	Spain: Pyrenees, Tobacor	4x	40669	SAMN14647068	MT145300
*Festuca pyrogea*	Argentina: Tierra de fuego	?	16835	SAMN14647069	MT145302
*Festuca quadridentata*	Ecuador: Chimborazo	?	15091	SAMN14647070	MT145303
*Festuca spectabilis*	Bosnia-Hercegovina: Troglav	6x	12960	SAMN14647071	MT145304
*Festuca superba*	Argentina: Jujuy, Yala	8x	12193	SAMN14647072	MT145305
*Festuca triflora*	Morocco: Rif, Ketama	2x	24472	SAMN14647073	MT145306
*Megalachne berteroniana*	Chile: JuanFernandez, Masatierra	?	5288	SAMN14647074	MT145307
*Megalachne masafuerana*	Chile: JuanFernandez, Masafuera	?	6134	SAMN14647075	MT145308
*Podophorus bromoides*	Chile: JuanFernandez, Masatierra	?	6694	SAMN14668162	——
*Vulpia ciliata*	Spain: Mar de Ontígola	4x	11801	SAMN14647076	MT145309
*Vulpia sicula*	Italia: Sicilia, Madone	2x	11327	SAMN14647077	MT145310
Outgroups					
*Brachypodium distachyon*	Iraq: near Salakudin	2x	-	NC_011032.1	phytozome.jgi.doe.gov, Bd21 v.3.1
*Oryza sativa* subsp. *japonica*	cv. PA64S; cv. Nipponbare	2x	-	AY522331.1	AP008215

### DNA Extraction and Sequencing

The 36 samples used in this study were obtained from herbarium specimens (AARHUS, K, MO, US, OS, CONC, HUTPL, University of Zaragoza), silica gel dried leaf tissues collected in field trips, and fresh leaves collected from plants growing in the Universidad de Zaragoza – Escuela Politécnica Superior de Huesca common garden (Table 1 and Supplementary Table S1). Total DNA from fresh and silica gel dried samples was isolated following the DNeasy Plant Mini kit (Qiagen, Valencia, CA, United States) protocol using 20–30 mg of dry leaf tissue or 20 mg of fresh tissue ground to powder with liquid nitrogen. Total DNA from herbarium samples was extracted using a modified CTAB protocol (Doyle and Doyle, 1987) using ∼20 mg of tissue. DNA concentration was quantified with a Qubit fluorometer (Invitrogen by Life Technologies, Carlsbad, California, United States) and DNA quality was evaluated with Biodrop (Harvard Bioscience). The integrity of the DNA was further checked in a 1% agarose gel. Overall the qualities and quantities of the DNAs were appropriate for genome skimming (∼5 μg, 50 ng/μl), except that of *P. bromoides*, which had <1 ng/μl.

DNAs obtained from three *Megalachne* and *Podophorus* samples plus 33 Loliinae samples were used to construct a genomic library for shotgun sequencing using Illumina technology. The library from freshly and herbarium collected materials DNAs was prepared with KAPA Hyper Prep Kit for PCR-free workflows (Roche Kapa Biociences) with some minor modifications. In brief, 1.0 μg of genomic DNA was sheared in a Covaris^TM^ E220 focused-ultrasonicator into Covaris microTUBE AFA Fiber Pre-Slit Snap-Cap tubes with the following parameters: sample volume 55 μl, duty cycle 15%, intensity 450, cycles/burst 200, time 100 s, temperature 4°C, in order to reach the fragment sizes of ∼200–400 bp. The sheared DNA was end-repaired, adenylated and ligated to IDT adaptors with unique dual-matched indexes (Integrated DNA Technologies) for paired end sequencing. The adaptor-modified end library was size selected and purified with AMPure XP beads (Agencourt, Beckman Coulter) in order to eliminate non-ligated adapters and adapter dimers. Final library size was confirmed on an Agilent 2100 Bioanalyzer with the DNA 7500 assay. The *Podophorus bromoides* library yielded 13 ng/μl and two normally distributed fragment size distributions of 200 and 500 bp. The PCR free library was quantified by Library Quantification Kit for Illumina Platforms (Roche Kapa Biosystems). The library was multiplexed with other libraries and the pool of libraries was then partly sequenced on a HiSeq4000 and partly on a HiSeq 2500 (TruSeq SBS Kit v4, Illumina, Inc) in paired-end mode (2 × 100 bp) in the Centro Nacional de Análisis Genómicos (CNAG, Barcelona). Primary data analysis, image analysis, base calling and quality scoring of the run were processed using the manufacturer’s software Real Time Analysis (RTA 2.7.7) for HiSeq4000, and RTA1.18.66.3 when using HiSeq2500, followed by generation of FASTQ sequence files.

Additionally, four Loliinae samples (Supplementary Table S1) were used for Sanger sequencing of the nuclear ribosomal ITS locus and the plastid *trn*LF and *trn*TL loci using the primers and procedures indicated in Inda et al. (2008) in Macrogen and were added to the 97 Loliinae data set obtained from previous studies (Inda et al., 2008; Minaya et al., 2017).

### DNA Sequence Data Assembling and Multiple Sequence Alignments

Illumina paired-end (PE) reads of the Fernandezian and other Loliinae samples were checked using FASTQC^2^ and the adapters and low quality sequences were trimmed using TRIMMOMATIC (Bolger et al., 2014) at the CNAG. Plastome assembly was performed with Novoplasty v.2.7.1 (Dierckxsens et al., 2017) using the published plastomes of *Festuca ovina* (JX871940.1) for fine-leaved taxa and of *F. pratensis* (JX871941) for broad-leaved taxa (Hand et al., 2013) as reference, and the following parameters: k-mer: 27 or 39, insert size: ∼300 bp, genome range: 120,000–220,000 bp, and PE reads: 101 bp. Assembled plastomes were aligned using MAFFT v.7.031b (Katoh and Standley, 2013) followed by visual inspection using Geneious R11^3^. Because Novoplasty failed to assemble the whole plastome of *P. bromoides* due to the low number and quality of total PE reads, we used a Geneious mapping and readmerging strategy to map its reads to three phylogenetically close plastomes (*Megalachne berteroniana*, *M. masafuerana*, *Festuca pampeana*).

For the assembly of the nuclear ribosomal cistron we used a two-step read mapping and merging approach. Due to the lack of any published Loliinae rDNA cistron, we employed the *Brachypodium distachyon* rDNA cistron (reference genome Bd21, Vogel et al., 2010)^4^ as reference and mapped to it the PE reads of the studied Loliinae taxa. Readmerging allowed us to align reads and their reverse complements to create a single consensus read. This step also allowed improving the sequence quality of overlapping parts. In cases of non-overlapping PE reads, the reads were used independently. The integrity of the cistron locus was examined visually for read mappings using Geneious R11.

Forward and reverse ITS, *trn*LF, and *trn*TL Sanger sequences were checked, corrected and merged using Sequencher v. 5.4.6 (Gene Codes Corporation, Ann Arbor, MI, United States)^5^. Each data set was aligned separately, visually inspected using Geneious R11 and manually corrected if necessary. The assembly of the *P. bromoides trn*LF and *trn*TL loci was done through several read mapping iterations with Geneious using as reference the closest *M. berteroniana*, *M. masafuerana*, *F. ventanicola* and *F. pampeana trn*LF and *trn*TL sequences.

A multiple sequence alignment (MSA) of 35 newly assembled *Megalachne* and Loliinae plastomes with *Oryza sativa* (AY522331.1; Genbank) and *Brachypodium distachyon* (NC_011032.1; Genbank) outgroups was performed with MAFFT v.7.215 (Katoh and Standley, 2013). The length of this full Loliinae plastome MSA without *Podophorus* was 146,172 bp length. The short *P. bromoides* consensus plastid sequence was subsequently aligned to the Loliinae full plastome MSA in Geneious R11. The multiple plastome alignment was filtered to remove poorly aligned regions and missing data in *P. bromoides* and other taxa through the automated option of trimAl v.1.2rev59 (Capella-Gutiérrez et al., 2009). The length of the filtered Loliinae plastome MSA with *Podophorus* was 55,872 bp length. A nuclear MSA of 35 newly assembled *Megalachne* and Loliinae rDNA cistrons and of *Oryza sativa* (AP008215; Genbank) and *Brachypodium distachyon* (Bd21; Phytozome) outgroups was also conducted with Geneious R11, rendering a 6,455 bp alignment. Independent MSAs were also produced for the ITS, *trn*LF, and *trn*TL loci of 135 Loliinae species and 16 outgroups, which included the *Megalachne* and *Podophorus* samples in Geneious R11. The *trn*LF and *trn*TL plastid loci were combined into a single plastid TLF MSA; separate phylogenetic analyses of the two loci gave congruent topologies with that recovered for the concatenated TLF haploid data matrix and only results from the latter analysis will be explained further. The nuclear ITS and the plastid TLF data set were further combined into a ITS-TLF MSA after obtaining congruence results from contrasted topological tests.

### Phylogenetic Reconstruction and Divergence Time Analysis

Maximum likelihood phylogenetic analysis of the plastome (full and reduced), the rDNA cistron, and the independent and combined ITS, and TLF data sets were conducted with IQTREE (Nguyen et al., 2015) imposing the best-fit nucleotide substitution model to each separate data set that was automatically selected by the ModelFinder option of the program (Kalyaanamoorthy et al., 2017) according to the Bayesian Information Criterion (BIC) [plastome (full and reduced): TVM + F + R3; rDNA cistron: GTR + F + R2; ITS: SYM + I + G4; TLF: K3Pu + FR4]. Each search was performed through the automated computation of 20 Maximum Likelihood (ML) starting trees from 98 alternative randomized Maximum Parsimony (MP) trees, searching for best-scoring ML trees and estimating branch support for the best tree from 1,000 bootstrap replicates (BS) using the ultrafast bootstrap option (Minh et al., 2013; Chernomor et al., 2016) implemented in the software.

Ancestral divergence ages of the Fernandezian and other Loliinae grasses were estimated from the concatenated ITS-TLF data set with BEAST 2 (Bouckaert et al., 2014). We imposed independent site substitution models, lognormal relaxed clock and Yule tree models (Minaya et al., 2017). Two nodes of the Poaceae tree were calibrated using secondary age constrains for the crown nodes of the BOP clade (*Oryza* + Pooideae) (normal prior mean = 51.9 Ma, *SD* = 1.9) and the *Brachypodium* + core pooids clade (*Brachypodium* + Aveneae-Poeae) (normal prior mean = 30.9 Ma, *SD* = 3.5), following the grass-wide plastome based dating analysis of Sancho et al. (2018) and a third node was calibrated using a minimum age constrain (16 Ma) for the crown node of the fine-leaved Loliinae (lognormal prior mean = 19.5 Ma, *SD* = 0.101) based on a *Festuca* leaf macrofossil from Poland dated to the Early Miocene showing *Festuca* sect. *Festuca*-type adaxial and abaxial epidermises (Juchniewicz, 1975). We also imposed a broad uniform distribution prior for the uncorrelated lognormal distribution (ucld) mean (lower = 1.0E-6; upper = 0.1) and an exponential prior for ucld standard deviation (SD). We ran 600 million Markov chain Monte Carlo (MCMC) generations in BEAST2 with a sampling frequency of 1,000 generations. The adequacy of parameters was checked using TRACER v.1.6^6^ with all the parameters showing Effective Sample Size (ESS) >200. A Maximum clade credibility (MCC) tree was computed after discarding 10% of the respective saved trees as burn-in.

### Ancestral Range Estimation

We used the parametric dispersal-extinction-cladogenesis (DEC) approach implemented in Lagrange v. 20130526 (Ree and Smith, 2008) to infer global extinction and dispersal rates and ancestral range inheritance scenarios for each node representing the ancestors of the Fernandezian and other Loliinae grasses in the maximum clade credibility (MCC) tree obtained from BEAST. We defined 13 Operational Areas (OAs) (A-M), selected according to the current distribution ranges of the species and the potential historical distributions of their ancestors, delimited by geographical features that could have acted as barriers to dispersal (Minaya et al., 2017) (Supplementary Table S2A). Specifically, we selected four American OAs: North America (H), northern South America (I), southern South America (J), and Juan Fernandez (M), aiming to recover the areas of origin of the ancestors of *Megalachne* and *Podophorus* that presumably colonized the Juan Fernandez archipelago from the American mainland through long-distance dispersal (LDD). The ancestral ranges were built imposing a maximum of two ancestral areas (AA), considering that ancestors were not more widespread than their extant descendants (Sanmartín, 2003). Ancestral range inheritances and biogeographic events were inferred from a stratified model with four temporal windows (TSI: Late Oligocene to Middle Miocene, 28.4–16.0 Ma; TSII: Middle to Late Miocene, 16.1–7.2 Ma; TSIII: Late Miocene to Pliocene, 7.3–2.6 Ma; TSIV: Quaternary, 2.61–0 Ma). This model included the different temporal paleogeographical configurations of the Americas and other continents that might have affected the evolution and the distribution of the main Fernandezian and other Loliinae lineages (Supplementary Table S2B). In order to obtain a more detailed fine-scale reconstruction of the biogeographic events that resulted in the Fenandezian grasses, a second DEC analysis was performed for the lineages of the American-Vulpia-Pampas clade where the Fernandezian subclade was nested within (see section Results). This second analysis was performed using a pruned MCC dated subtree for the American-Vulpia-Pampas clade and five OAs representing the current and paleo-geographical distributions of the lineages (H-North-Central America, N-Pampas-Ventania, O-Andes, P-Masatierra, Q-Masafuera; Supplementary Tables S2C,D).

## Results

### Loliinae Genome Sequence Data, Plastomes, and Nuclear rDNA Cistrons

Most of the studied Loliinae genome-skimming sequenced samples, including the newly studied *Festuca asplundii*, *F. caldasii*, *F. holubii*, *F. procera*, and *F. quadridentata*, yielded a large number of PE reads, ranging from 9,863 to 40,669 kbp (Table 1 and Supplementary Table S1). The two *Megalachne* samples were below that threshold (*M. berteroniana* 5,288 kbp; *M. masafuerana* 6,134 kbp) but showed high quality reads. The 164 years old *Podophorus bromoides* type specimen sample rendered 6,694 kbp poor quality PE reads (Table 1 and Supplementary Table S1).

Most plastid assemblies produced a single plastome contig with a deep coverage of >50x per sample that contained its two inverted repeat regions (IRa, IRb). However, Novoplasty assemblies of *Festuca durandoi, F. spectabilis, F. superba*, *F. molokaiensis*, *F. abyssinica*, and *Megalachne berteroniana* gave several small contigs and their full plastome assemblies were constructed with these contigs and the read mapping approach using Geneious and plastomes of their closest species as references. Plastome lengths of broad-leaved Loliinae ranged from 134,231 to 134,734 bp and those of fine-leaved Loliinae from 132,599 to 133,869 bp; these values agreed with the plastome lengths retrieved by Hand et al. (2013) for their two main Loliinae group taxa. The lengths of the *Megalachne berteroniana* (132,812 bp) and *M. masafuerana* (132,826 bp) plastomes fell within the fine-leaved Loliinae range. The PE reads of the newly assembled plastomes were deposited in GeneBank under BioProject PRJNA626668^7^ with accessions numbers SAMN14647043–SAMN14647077 and SAMN14668162 (Table 1 and Supplementary Table S1). The full Loliinae plastome MSA is available in Github^8^. The *Podophorus bromoides* plastid consensus sequence (total length ∼69,238 bp) covered different non-overlapping fragments of the aligned Loliinae plastomes (∼40.7%) with a low coverage depth (10x to 1x). The plastid *P. bromoides* sequence (with its nucleotide positions mapped against the full Loliinae plastome MSA) is available in Github^9^.

We obtained a single contig of 6,453–6,455 bp for the rDNA cistrons of the studied *Megalachne* and other Loliinae samples. Coverage depth was relatively constant across the rDNA cistron sequences in most cases (>10x). The newly sequenced rDNA cistrons were deposited in GeneBank with accessions numbers MT145276–MT145310 (Table 1). The low quality genomic sequence available in the DNA obtained from the *P. bromoides* specimen resulted in a low number of PE reads, which precluded the readmerging of its full rDNA cistron; however, it allowed the assembly of its entire ITS region (Table 1 and Supplementary Table S1). The nuclear rDNA cistron of the studied *Megalachne* and other Loliinae grasses had a conserved structure along its transcriptional unit of 6–6.5 kb length, containing the 5’-ETS (724 bp), the 18S gene (1,818 bp), the ITS (585 bp), and the 25S gene (3,408 bp) regions of similar mean length to those of other grasses.

The nuclear ITS locus and the plastid *trn*LF and *trn*TL loci were filtered, respectively, from the assembled rDNA cistrons and plastomes for the *Megalachne* and Loliinae samples (Table 1 and Supplementary Table S1). For *P. bromoides*, the complete ITS sequence was recovered with a coverage depth ranging from 10x to 1x and was deposited in Genbank under accession code MT022522 (Supplementary Table S1). Up to 60 and 70% of, respectively, the *P. bromoides trn*LF and *trn*TL sequences were recovered with a coverage depth of 10x (MSAs available in Github) (see footnote 9). The ITS and TLF sequences of the newly analyzed *F. andicola*, *F. longipes*, *F. vaginalis*, and *F. valdesii* were incorporated to the study and were deposited in Genbank under accession codes EF584922-EF592955-EF585009; KY368804-KY368856-KY368907; EF584977-EF584977-EF585111; MT022522-MT040974 – MT040975 (Supplementary Table S1).

### Loliinae Plastome and Nuclear Phylogenomic Trees

**FIGURE 2:**
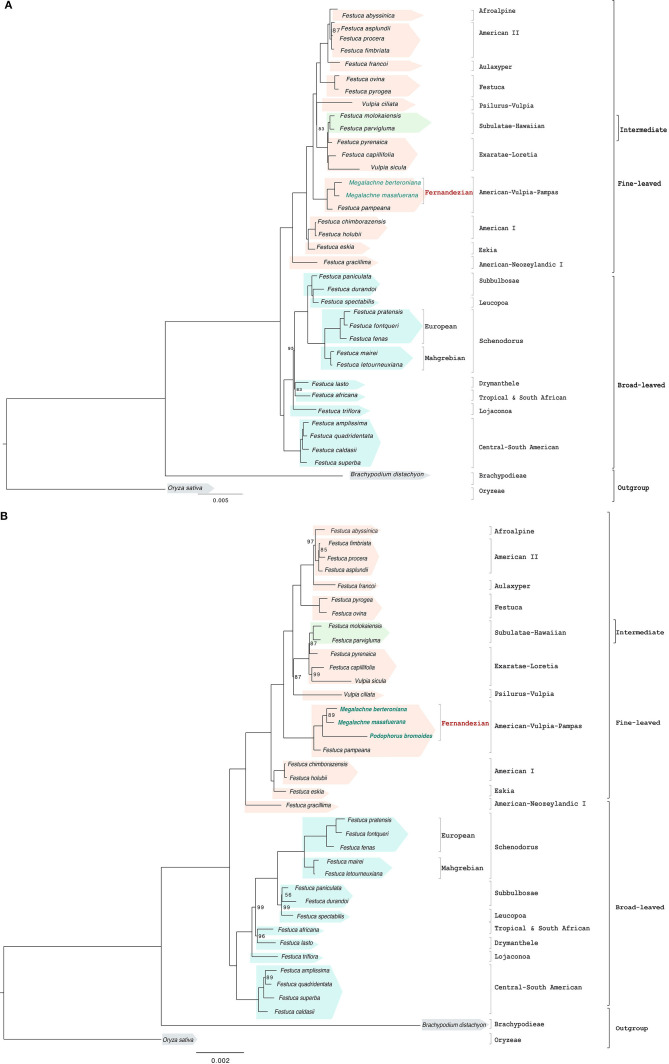
Maximum likelihood full plastome **(A)** and reduced plastome **(B)** trees constructed with IQTREE showing the relationships among the studied Fernandezian and Loliinae grasses. *Oryza sativa* was used to root the trees. Numbers indicate branches with UltraFast Bootstrap supports (BS) <100%; the remaining branches have 100% BS value.

The full plastome data set (two *Megalachne* and 33 additional Loliinae samples) included 133,894 filtered positions of which 7,480 were variable and 4,160 potentially informative. The best plastome ML phylogenetic tree (Figure 2A and Supplementary Figure S1A) recovered a fully resolved and highly supported topology with most branches having 100% bootstrap support (BS), and only three (94–99% BS) and one (77% BS) branches having strong to relatively good support. This Loliinae phylogenomic tree based on plastome data showed a main split of broad vs. fine-leaved Loliinae lineages, and successive splits within both the broad-leaved (Central-South American, Lojaconoa, Drymanthele/Tropical and South African, Leucopoa, Subbulbosae, Schedonorus) and the fine-leaved (American-Neozeylandic I, Eskia/American I, American-Vulpia-Pampas, Psilurus-Vulpia/Exaratae-Loretia (with intermediate Subulatae-Hawaiian nested within), Festuca, Aulaxyper, American II, Afroalpine) clades. *Megalachne berteroniana* and *M. masafuerana* plastome sequences formed a Fernandezian clade, sister to *F. pampeana* and nested within the southern American American-Vulpia-Pampas clade. Newly sequenced South American plastome samples fell within the fine-leaved American II [*F. fimbriata*, (*F. asplundii*, *F. procera*)] and American I [(*F. holubii*, *F. chimboracensis*)] clades, and within a Central-South American broad-leaved clade [(*F. caldasii*, (*F. superba*, (*F. quadridentata*, *F. amplissima*)))]. Fuegian *F. pyrogea* fell within the fine-leaved Festuca clade and the broad-leaved *F. fenas* clustered within the European Schedonorus clade (Figure 2A and Supplementary Figure S1A).

The reduced plastome data set, which included the *Podophorus* sample, had 55,872 positions of which 5,989 were variable and 823 potentially informative. The optimal ML tree (Figure 2B and Supplementary Figure S1B) recovered a topology that was also fully resolved and almost identical to that of the complete plastome data, though the branch support was slightly lower across the phylogenomic tree [all braches with full support except seven branches with strong (90–99%), three with good (70–89%), and one with weak (60%) BS]. In this phylogenomic tree, *P. bromoides* was resolved as sister to the *Megalachne* subclade (90% BS) and formed a fully supported Fernandezian clade, which was nested within the American-Vulpia-Pampas lineage (Figure 2B and Supplementary Figure S1B).

**FIGURE 3:**
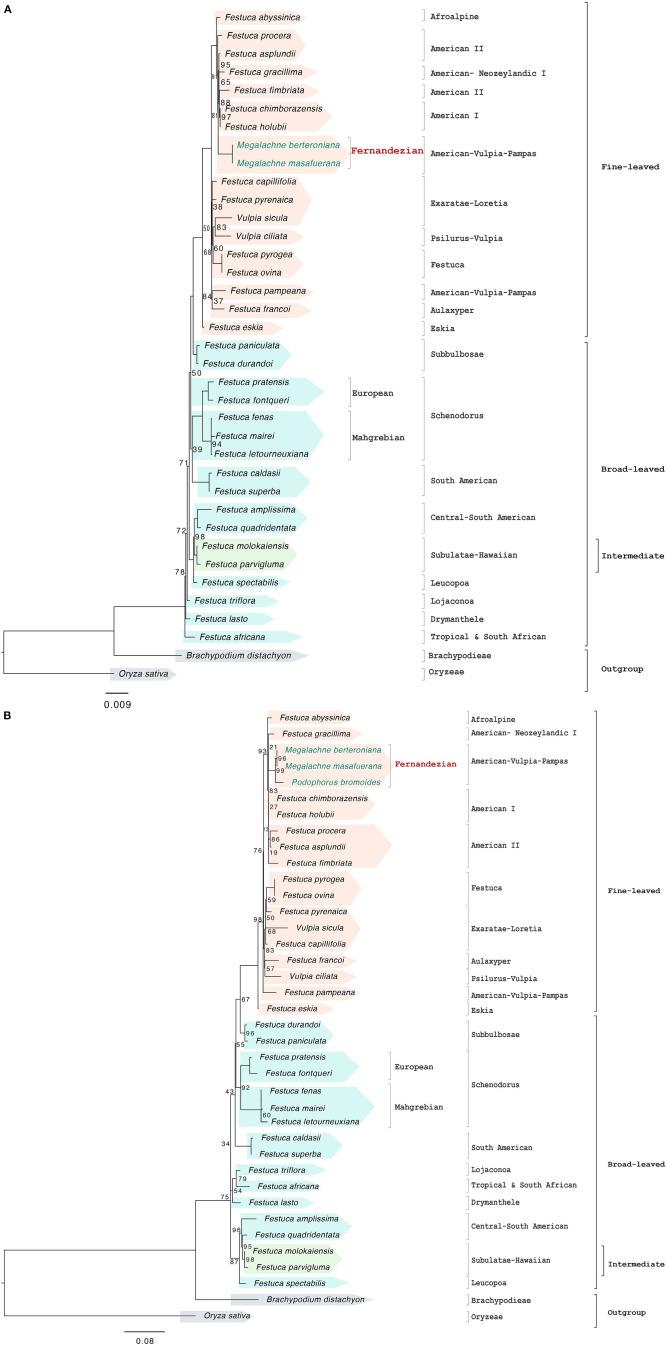
Maximum likelihood nuclear rDNA cistron **(A)** and ITS **(B)** trees constructed with IQTREE showing the relationships among the studied Fernandezian and Loliinae grasses. *Oryza sativa* was used to root the trees. Numbers indicate branches with UltraFast Bootstrap supports (BS) <100%; the remaining branches have 100% BS value.

The nuclear rDNA cistron data set (two *Megalachne* and 33 additional Loliinae samples) included 6,455 positions of which 502 were variable and 321 potentially informative. The best ML tree (Figure 3A and Supplementary Figure S1C) retrieved a fully resolved topology; however, some internal branches were very short and showed very low support [21 branches with strong (90–99%), seven with good (70–89%), and seven with weak (60%) or very weak (<50%) BS]. The rDNA cistron-based phylogenetic tree showed the successive divergences of early diverging paraphyletic broad-leaved lineages (Tropical and South African, Drymanthele, Lojaconoa, Leucopoa, Central-South American, South-American, Schedonorus, Subbulbosae), which were in most cases poorly supported and included the intermediate Subulatae-Hawaiian nested within, and the more recent split of the strongly supported fine-leaved clade (97% BS). The topology of the fine-leaved group showed successive weakly to strongly supported lineage splits [(Eskia, ((Aulaxyper, Exaratae-Loretia, Festuca), (American-Vulpia-Pampas, Psilurus-Vulpia, Afroalpine, American-Neozeylandic I, American I, American II)))]. *Megalachne berteroniana* and *M. masafuerana* formed a fully supported Fernandezian clade based on the cistron sequences; this clade was close to other species of the American I (*F. holubii*, *F. chimborazensis*) and American II (*F. asplundii*, *F. fimbriata, F. procera*) assemblages, which together with the American-Neozeylandic I *F. gracillima* formed a well-supported fine-leaved South American clade (91% BS). *Festuca pyrogea* was reconstructed as sister to *F. ovina* within the strong Festuca clade. Within the broad-leaved lineages, the strongly supported Central-South American (*F. amplissima*, *F. quadridentata*) and (*F. superba*, *F. caldasii*) clades were resolved in different positions across the broad-leaved subtree, and *F. fenas* clustered within the Mahgrebian Schedonorus subclade (Figure 3A and Supplementary Figure S1C). Phylogenetic reconstruction of filtered rDNA cistron sequences for the ITS region, together with that of *P. bromoides*, recovered the same overall tree topology, which showed a strong sister relationship of *P. bromoides* to the *Megalachne* clade (99% BS) (Figure 3B and Supplementary Figure S1D).

### Plastid TLF, Nuclear ITS, and Combined ITS-TLF Phylogenetic Relationships

The separate and combined TLF (2,205 positions, 501 variable, 240 informative), ITS (645 positions, 285 variable, 193 informative) and ITS-TLF analyses of 135 Loliinae and outgroup samples retrieved phylogenies (Supplementary Figures S2A–C) highly congruent with those obtained in previous studies. Additionally, these trees showed the evolutionary placements of the three Fernandezian species and of six South American and one South African newly studied *Festuca* taxa. Both the nuclear ITS and the plastid TLF recovered a highly supported Fernandezian clade (99% BS) where *P. bromoides* was sister to the *M. berteroniana*/*M. masafuerana* subclade. Nonetheless, whereas the Fernandezian group was nested within a clade of American-Vulpia-Pampas taxa (69% BS), clearly separated from the American I (82% BS), and American II+Afroalpine (78% BS) clades in the TLF tree (Supplementary Figure S2A), it was nested within a large clade of American I + American II + Afroalpine taxa (99%) that also included some (*F. ventanicola*) but not all the American-Vulpia-Pampas species in the ITS tree (Supplementary Figure S2B). The combined ITS-TLF analysis placed the fully supported Fernandezian clade within a highly supported American-Vulpia-Pampas clade (97% BS) and resolved *F. ventanicola* as the strong sister lineage of the Fernandezian grasses (100% BS) (Supplementary Figure S2C). The TLF and ITS evolutionary placements of the newly sequenced South American taxa agreed with those of the plastome and rDNA trees and were overall congruent to each other. The fine-leaved *F. asplundii* and *F. procera* were nested within the American II + Afroalpine clade and *F. holubii* within the American I clade in the TLF tree (Supplementary Figure S2A), whereas the three of them fell within the large American I + American II + Afroalpine clade in the ITS tree (Supplementary Figure S2B). The sister *F. asplundii*/*F. andicola* (69% BS) and *F. holubii*/*F. glumosa* (87% BS) relationships observed in the ITS tree and their phylogenetic placements in the combined ITS-TLF tree (Supplementary Figure S2C) agreed with those of the plastid tree. The broad-leaved *F. quadridentata* and *F. caldasii* were nested within a large Central-South American-Eurasian-South African clade (97% BS) in the TLF tree (Supplementary Figure S2A) and in separate Central-South American (74% BS) and Eurasian-South American (62% BS) clades in the ITS tree (Supplementary Figure S2B). Their positions in the combined ITS-TLF tree (Supplementary Figure S2C) agreed with those of the nuclear tree. The South African *F. longipes* was resolved as sister of South African *F. scabra* (99% BS) in the TLF tree (Central-South American-Eurasian-South African clade) and of Tropical-South African *F. costata* in the ITS (100% BS) and combined ITS-TLF (88% BS) trees (Tropical-South African clade) (Supplementary Figures S2A–C).

### Dating Analysis and Ancestral Range Inheritance Reconstruction

**FIGURE 4:**
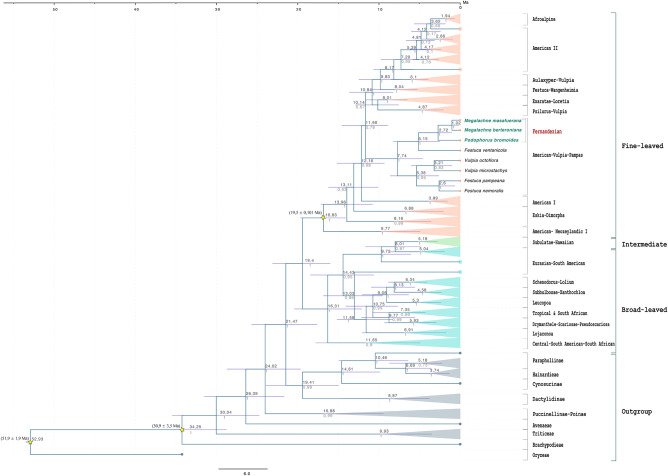
Schematic Bayesian maximum clade credibility dated chronogram of 135 Loliinae taxa constructed with BEAST2 using nuclear ITS and plastid TLF loci showing estimated nodal divergence times (medians, in Ma) and 95% highest posterior density (HPD) intervals (bars) above branches and Posterior Probability Support (PPT) values below branches. Stars indicate secondary nodal calibration priors (means ± SD, in Mya) for the crown nodes of the BOP, *Brachypodium* + core pooids, and fine-leaved Loliinae clades.

The Bayesian ITS-TLF MCC tree constructed with Beast2 (Figure 4 and Supplementary Figure S3) yielded a similar topology to that retrieved in the ML analysis (Supplementary Figure S2C). The age of stem and crown Loliinae nodes were estimated to Late-Oligocene (median 21.47 Ma) and Early Miocene (19.4 Ma), respectively, whereas Early and Mid-Miocene divergences were inferred for the splits of the broad (16.31 Ma) and fine-leaved (16.83 Ma) lineages. An older Mid-Miocene origin was estimated for the ancestor of the American-Vulpia-Pampas clade (7.74 Ma) than for the younger Late-Miocene-to-Pliocene ancestors of the remaining fine-leaved [American II+Afroalpine (5.39 Ma); American I (3.89 Ma)] and broad-leaved [South-American (5.04 Ma); Central-South American (3.32 Ma)] South American Loliinae lineages (Figure 4 and Supplementary Figure S3). The ancestor of the Fernandezian clade was inferred to have originated between the Late-Miocene (5.15 Ma; stem node) and the Pliocene (2.72 Ma; crown node), corresponding to the estimated split of *Podophorus* and *Megalachne*, whereas the split of the two *Megalachne* species was estimated to have occurred in the Pleistocene (1.02 Ma, Calabrian). The estimated ages of the Fernandezian ancestors predated those inferred for the ancestor of other oceanic endemic Loliinae lineages [e.g., Canarian fine-leaved Aulaxyper (4.11–2.84 Ma; Pliocene); Hawaiian *F. aloha*/*F. molokaiensis* (1.89–1.16 Ma; Lower-to-Recent Pleistocene); and recent Pleistocene Madeiran broad-leaved *F. donax* (1.23 Ma, Calabrian) and Reunion Island fine-leaved *F. borbonica* (0.3 Ma, Ionian)] (Figure 4 and Supplementary Figure S3).

**FIGURE 5:**
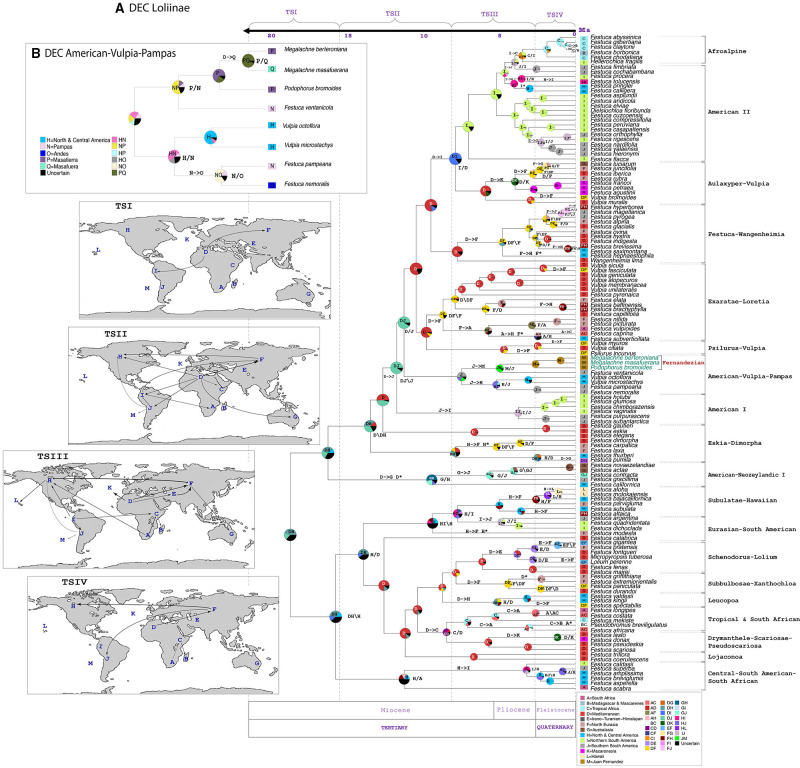
Estimated ancestral ranges and biogeographical events of the Fernandezian and other Loliinae grasses inferred from LAGRANGE under the stratified DEC models mapped on the BEAST2 maximum clade credibility tree with outgroups pruned from it. **(A)** Loliinae DEC model; **(B)** American-Vulpia-Pampas DEC model. The vertical dashed lines separate the four time slices (TSI-TSIV) used in the Lagrange analyses. The maps on the left represent the palaeogeographical configuration of the world in these four time periods and the arrows represent the dispersals between areas that reflect change in continental connectivity over time. The pie charts at the nodes indicate the relative probabilities for alternative ancestral ranges (with their color legends indicated at the respective inset charts). The inferred biogeographic events are indicated at the nodes (X/Y vicariance; X\Y peripheral isolation) and branches (X->Y dispersal; X* extinction) of the tree. The Operational Areas assigned to the species are indicated to the right of the trees.

The ancestral range inheritance scenarios of Loliinae inferred from our Lagrange stratified Loliinae DEC model (-ln likelihood 404.6) had a global estimated dispersal rate (*dis*: 0.09385) 5.5 times higher than the estimated extinction rate (*ext*: 0.01536) (Figure 5A). The ancestors of Loliinae and of the broad-leaved and fine-leaved clades were inferred to have originated in uncertain widespread areas of the northern hemisphere (Mediterranean basin, Northern-Central America, Eurasia) in the transition between the Late Oligocene and the Early Miocene. Most of the transcontinental LDDs of both fine-leaved and broad-leaved Loliinae ancestors were estimated to have occurred during the Miocene and the Pliocene (time slices TSII-TSIII), and a few more during the Pleistocene (time slice TIV) (Figure 5A). According to our DEC model, the South American subcontinent was simultaneously colonized by broad and fine-leaved Mediterranean ancestors, which arrived, respectively, to the northern and southern South American ranges around the Mid-Miocene (Figures 4, 5A). Within the fine-leaved lineage, a Mid-Miocene vicariance was inferred to have originated the American-Vulpia-Pampas ancestor in southern South America ∼7.74 Ma. This ancestor would have then experienced range expansions to either North-Central America originating the southern American Pampean-Andean and the North-Central American Vulpia clade and to the Juan Fernandez archipelago originating the Pampean-Fernandezian clade at the end of the Neogene. Our stratified Loliinae DEC model suggested that the colonization of the Juan Fernandez archipelago from a mainland ancestor in southern South America could have occurred in the Mid-to-Late Miocene (7.74–5.15 Ma) (Figures 4, 5A). According to this hypothesis, the ancestor of *F. ventanicola* and the Fernandezian *Podophorus* and *Megalachne* grasses was distributed in a widespread southern South America-Juan Fernandez area during the Late Miocene (5.15 Ma). A vicariance event was invoked to explain the split of the common ancestor into the current mainland Pampean-Ventanian endemic lineage and the Fernandezian ancestor, which was inferred to be present in the archipelago in the mid-Pliocene (2.72 Ma) (Figures 4, 5A). A more detailed reconstruction of the biogeography of the Fernandezian grasses within their archipelago was obtained in our second American-Vulpia-Pampas DEC model (-ln likelihood 406.2; dis: 0.08232; ext: 0.0497) (Figure 5B). According to this model: (i) the ancestor of the American-Vulpia-Pampas could have been distributed in the Pampas-Ventanian range during the Miocene (7.74 Ma); (ii) this ancestor presumably experienced a range expansion to Masatierra and was present in a widespread Pampas-Fernandezian area during the Late Miocene (5.15 Ma); (iii) after a Pampas-Ventanian/Masatierra vicariance, the ancestor of the Fernandezian grasses was present in Masatierra during the Late Pliocene (2.72 Ma); (iv) an *in situ* speciation event originated the *Podophorus* lineage in Masatierra at that time; (v) a range expansion from Masatierra to Masafuera placed the ancestor of the *Megalachne* clade in the two main Juan Fernandez islands during the Pleistocene (1.02 Ma); (vi) a recent vicariance would explain the respective speciations of *M. berteroniana* in Masatierra and of *M. masafuerana* in Masafuera during the last million years (Figures 4, 5B).

## Discussion

### Phylogenetics of *Megalachne* and *Podophorus*: The Loliinae Fernandezian Clade

Our museomic approach, based on the combined use of old and recent herbarium samples and of genome skim data, have allowed us to disentangle the evolutionary origins of the neglected *Megalachne* and *Podophorus* grasses. Complete and partial plastomes as well as the nuclear rDNA cistron and ITS data supported the phylogenetic placement of the studied Fernandezian *Podophorous bromoides*, *Megalachne berteroniana*, and *M. masafuerana* species within the American-Vulpia-Pampas fine-leaved Loliinae clade (Figures 2, 3 and Supplementary Figures S1A–C). Our results corroborate the early suggestions of Tateoka (1962) who indicated a close affinity of *Megalachne* to *Festuca* based on shared morphological and serological traits, and the recent phylogenetic findings of Schneider et al. (2011, 2012) and Tkach et al. (2020) who placed them within the fine-leaved Loliinae, and definitively discard its classification within either Bromeae or Duthieinae. Our results have also contributed to enlarge the paraphyly of *Festuca*, which now accounts to up to 14 Loliinae genera nested within its main fine-leaved (*Ctenopsis, Dielsiochloa, Hellerochloa, Megalachne, Micropyrum, Narduroides, Podophorus, Psilurus, Vulpia, Wangeheimia*), intermediate (*Castellia*), and broad-leaved (*Lolium, Micropyropsis, Pseudobromus*) lineages (Supplementary Figure S2C; Inda et al., 2008; Minaya et al., 2017).

Our study has demonstrated the utility of museomics to disentangle the evolutionary history of the extinct *Podophorus bromoides* from its 164 years old type specimen. This adds a new extinguished species to the tree-of-life, resolving its phylogenetic position within the grasses, as done before for other exterminated plants, such as *Sicyos villosus* within Cucurbitaceae (Sebastian et al., 2010) *Hesperelaea palmeri* within Oleaceae (Van de Paer et al., 2016; Zedane et al., 2016), *Haplostachys linearifolia* and *Stenogyne haliakalae* within Lamiaceae (Welch et al., 2016) and *Chasechloa egregia* within Poaceae (Silva et al., 2017). Moreover, our phylogenetic analyses based on plastome and rDNA-based data have demonstrated that *P. bromoides* is strongly resolved as sister to the *Megalachne* clade (*M. berteroniana*, *M. masafuerana*) (Figures 2B, 3B and Supplementary Figures S2A–C), rejecting thus the moderately supported sister relationship found for the Masatierra taxa (i.e., *P. bromoides* and *M. berteroniana*, 72% BS) in a previous phylogenetic analysis based on partial ITS sequences from some samples (*Podophorus bromoides* ITS1 only) (Schneider et al., 2011).

Our Loliinae-wide phylogenomic analyses have further identified the relict Pampean-Ventanian fescues as the closest relatives of these fine-leaved endemic Fernandezian grasses. Phylogenies based on complete and partial plastome data indicate that *Megalachne* and *Podophorus* are strongly related to the American-Vulpia-Pampas lineage, represented by *F. pampeana* (Figure 2 and Supplementary Figure S2A). By contrast, the nuclear rDNA cistron and the ITS phylogenies place them within a large American I + American II group (Figure 3 and Supplementary Figure S2B), an assemblage that also includes other American-Vulpia-Pampas species, such as *F. ventanicola* (Supplementary Figure S2B). However, the phylogenetic tree reconstructed with the combined ITS-TLF data strongly supports nesting the Fernandezian clade within the American-Vulpia-Pampas clade and its sister relationship to the Pampean-Ventanian endemic *F. ventanicola* (Figure 4 and Supplementary Figures S2C, S3). The incongruent placements of the Fernandezian grasses in the maternal plastome (plastid) vs. paternal rDNA cistron (ITS) Loliinae trees is a general feature of many Southern Hemisphere Loliinae species that reflect their hybrid allopolyploid nature (Inda et al., 2008; Minaya et al., 2017). Evolutionary studies have illustrated the different topological placements of known allopolyploid Loliinae species in plastid vs. nuclear trees (e.g., allotetraploid *F. fenas*, allohexaploid *F. arundinacea*, Inda et al., 2014; allotetraploid *F. simensis*, Inda et al., 2014; Minaya et al., 2015; allohexaploid *F. nigrescens*, Kergunteuil et al., 2020). Karyological and genome size reports have further shown that all southern hemisphere Loliinae species studied so far are polyploids (Dubcovsky and Martínez, 1992; Connor, 1998; Namaganda et al., 2006; Smarda and Stancik, 2006). Therefore, the incongruent positions shown by the American I clade polyploids *F. chimborazensis* (4*x*), *F. vaginalis* (4*x*), *F. glumosa* (4*x*), *F. purpurascens* (6*x*), American-Vulpia-Pampas clade *F. ventanicola* (4*x*) and the putative South African polyploid *F. longipes* in our plastid and nuclear trees (Supplementary Table S1 and Supplementary Figures S2A,B) indicate that these taxa probably originated from interspecific hybridization followed by genome doubling. Although genome size or chromosome counting data are lacking for the Fernandezian *P. bromoides* and *M. berteroniana* and *M. masafuerana* species, their equivalent contrasting positions in the plastid and nuclear trees suggest that these endemic grasses are also allopolyploids. It is further supported by the fact that most of the remaining members of the American-Vulpia-Pampas clade are also polyploids [e.g., *F. pampeana* (8*x*), *F. nemoralis* (8*x*), *V. microstachys* (6*x*); Dubcovsky and Martínez, 1992; Smarda and Stancik, 2006; Díaz-Pérez et al., 2014]. Further investigation of these genomic data using the methodology described in Viruel et al. (2019) together with customized genome size analyses from fresh or herbarium samples (Smarda and Stancik, 2006) might reveal the ploidy level of these rare taxa.

*Megalachne* and *Podophorus* show a “vulpioid” phenotype, having lax panicles and long awned lemmas (Figure 1). These are characteristic traits of *Vulpia* and few other Loliinae lineages (Catalan et al., 2007). *Vulpia* and other ephemeral Loliinae genera, such as *Ctenopsis*, separate from *Festuca* based on their annual habit, four or less fertile florets per spikelet, largely unequal glumes, and long awned lemmas, which together distinguish them from the typical festucoid phenotype of *Festuca* and other robust Loliinae, characterized by their perennial habit, four or more fertile florets per spikelet, subequal glumes, and muticous or usually shortly awned lemmas, though none of them is absolute (Catalan et al., 2007). The origins of the polyphyletic *Vulpia* lineages are still intriguing although analysis of cloned single copy genes have demonstrated that some allopolyploid *Vulpia* species bear heterologous copies derived from morphologically close diploid relatives (Díaz-Pérez et al., 2014). The homoplasic “vulpioid” inflorescence phenotype has also appeared in other perennial Loliinae lineages, like the northern South America *Dielsiochloa floribunda* (American II clade), and in some species of *Festuca*. Interestingly, the slender cespitose Pampean-Ventanian endemic *F. ventanicola* shares its “vulpioid” phenotype with its sister Fernandezian *Megalachne* and *Podophorus* taxa (Figure 1), suggesting that they could have inherited it from their common ancestor. The long awn is an important dispersal trait in several annual grasses, including the invasive *Vulpia* species (Catalan et al., 2007; Díaz-Pérez et al., 2014), allowing the caryopsis to attach to the feathers or furs of animals and to be dispersed to long distances (Linder et al., 2018). It could be thus hypothetised that the presumed “vulpioid” ancestor of the Fernandezian grasses could have migrated to the isolated Juan Fernandez archipelago transported by epizoochory or endozoochory through pelagic birds. Interestingly, *Podophorus bromoides* shows an extremely reduced spikelet (Figure 1), being the only Loliinae taxon, together with *Vulpia fontquerana* Melderis & Stace (Torrecilla et al., 2004) having a single fertile floret (with a reduced sterile floscule) per spikelet. This, together with its apparent ephemeral habit might be associated to an overall trend toward an annual habit after its speciation in the Masatierra island (Figures 1, 5).

### Biogeography and Conservation of the Endemic *Megalachne* and *Podophorus* Grasses

Our Loliinae and American-Vupia-Pampas biogeographic DEC analyses have elucidated the most likely colonization routes of the Fernandezian ancestors, and the speciation events that originated *Podophorus* and *Megalachne* taxa in Masatierra and Masafuera (Figures 1, 5). Our ancestral range analyses identified the Pampean-Ventanian region to be the most likely place of origin for the common ancestors of the Fernandezian endemic grasses (Figures 5A,B). The closest relatives of *Podophorus* and *Megalachne* are relict endemic species of the Ventanian region (*F. ventanicola*, *F. pampeana*; Catalan and Müller, 2012) a hotspot of plant and animal diversity (Crisci et al., 2001). The formation of the Ventanian range in the Paleoproterozoic-Ordovician time span (∼2,200–475 Ma; Ramos et al., 2014) largely preceded the Oligocene-Miocene uplifting of the North American (31–28 Ma) and Central-Southern Andean (10–5 Ma) cordilleras (Crisci et al., 2001; Wakabayashi and Sawyer, 2001) as well as the emergence of the volcanic Juan Fernandez archipelago islands (5.8 Ma) (Stuessy et al., 1984). Although the inferred ages of the American-Vulpia-Pampas clade (7.7 Ma), *F. ventanicola* + Fernandezian clade (5.1 Ma) and Fernandezian clade (2.7 Ma) ancestors (Figure 4 and Supplementary Figure S3) are younger than those of the Central-Southern Andes, the altitude and disposition of the austral Andean mountains was probably lower than in the present (Crisci et al., 2001). The geological time layout could have facilitated the hypothetical LDDs of the Ventanian ancestors to other American ranges and to the Juan Fernandez archipelago (Figures 1, 5). Our Loliinae and American-Vulpia-Pampas DEC models support a colonization of the Fernandezian archipelago from a southern South American Pampean-Ventanian ancestor in the late-Miocene 7.7–5.1 Ma (Figures 4, 5). The most recent estimate for that colonization concurs with the radiometric dating of the oldest Fernandezian islands (Santa Clara, 5.8 ± 2.1 Ma; Masatierra, 4.23 ± 0.16 Ma) (Stuessy et al., 1984) which could have been united in the past (Sanders et al., 1987). We could thus infer that the Ventanian Fernandezian ancestors likely arrived at the paleo-island formed by Santa Clara and Masatierra during the Late Miocene (Figure 5), probably transported by birds. The estimated split of the *Podophorus* lineage from the *Megalachne* ancestor at 2.7 Ma suggest a late-Pliocene *in situ* speciation event in Masatierra for the origin of the endemic *P. bromoides* (Figure 4). Our regional DEC model and our dating analyses infer that the colonization of the Masafuera island occurred from Masatierra during recent Pleistocene times (1.02 Ma), supporting *in situ* speciation events for *M. berteroniana* in Masatierra and *M. masafuerana* in Masafuera (Figures 4, 5B). The westward inter-island colonization likely took place after the emergence of the young Masafuera island in the early Pleistocene (2.44 ± 1.14 Ma) (Stuessy et al., 1984) and was probably favored by the short distance separating them (i.e., 180 km, Figure 1). This distance has acted, however, as a strong geographic barrier to gene flow since the divergence of both species. Our biogeographic reconstruction for the Fernandezian Loliinae taxa agree with the hypothesis of higher levels of plant endemism in Masatierra compared to Masafuera, which are related to their respective distances to the closest mainland and their estimated ages (Stuessy et al., 2017). Our study has also identified the previously unknown South American ancestors of these endemic Fernandezian grasses, pointing to the relict Pampean-Ventanian region as their cradle (Figure 5B).

The rich endemic flora of Juan Fernandez archipelago is one of the most threatened on earth (Stuessy et al., 1998; Bernardello et al., 2006). Human impact on these islands, such as the introduction of environmentally aggressive herbivores, has probably caused the extinction of at least two endemic Fernandezian endemic plants during the last two centuries (*Santalum fernandezianum* Phil. and *Podophorus bromoides*; Bernardello et al., 2006; Danton et al., 2006). The latter extinct species was extremely rare; collected by Germain in 1854 and described by Philippi in 1856 from Masatierra (without a specific locotype), its existence was later mentioned by Johow in 1896 (Baeza et al., 2007). However, the plant was never seen again, even after exhaustive searches, and was therefore considered extinct (Stuessy et al., 1998, 2017; Baeza et al., 2007). All four *Megalachne* species are classified as threatened according to the IUCN categories of threat (Danton et al., 2006; Danton and Perrier, 2017; Penneckamp-Furniel, 2018; Penneckamp-Furniel and Villegas, 2019): *M. berteroniana* as Vulnerable, *M. masafuerana* as Endangered, *M. dantonii* as Critically Endangered, and *M. robinsoniana* as Endangered. Nonetheless, these IUCN assessments did not include a description of the employed IUCN criteria to classify the plants in their respective categories of menace. Several authors, however, have severe concerns about the threats posed to these endemic grasses by the introduced herbivores and by invasive plants (Stuessy et al., 1998; Bernardello et al., 2006; Danton et al., 2006; Danton and Perrier, 2017) and their survival in some inaccessible places to overgrazing pressure (Danton et al., 2006; Danton and Perrier, 2017). Rigorous populations censuses and population genetic studies of the more largely distributed *M. berteroniana* and *M. masafuerana* species, and of the recently described and still poorly known *M. robinsoniana* and *M. dantonii* species would be required to establish their adequate category of threat and to design appropriate conservation strategies. Historical collections have an enormous value for biogeographical studies. Several plants have gone to extinction in a few decades after human arrival due their high sensitivity to perturbation of their habitats and their low competitiveness, especially in oceanic islands (Sebastian et al., 2010; Van de Paer et al., 2016; Welch et al., 2016; Zedane et al., 2016; Silva et al., 2017). Regrettably, *Podophorus bromoides* sums up to the list of recently extinct plants although its museomic analysis has unveiled its historical biogeography.

## Data Availability Statement

All datasets generated for this study are included in the article/Supplementary Material.

## Author Contributions

PC designed the study. MM-A, IA, JV, and PC collected the samples. MM-A and JV developed the experimental work. PC, MM-A, JV, IA, and AS-R analyzed the data, interpreted the results, and revised the manuscript. PC and MM-A prepared the manuscript. All authors contributed to the article and approved the submitted version.

## Conflict of Interest

The authors declare that the research was conducted in the absence of any commercial or financial relationships that could be construed as a potential conflict of interest.

## Footnotes

1
https://apps.kew.org/herbcat/getImage.do?imageBarcode=K000433684

2
http://www.bioinformatics.babraham.ac.uk/projects/fastqc/

3
https://www.geneious.com

4 http://phytozome.jgi.doe.gov, v3.1Phytozome

5
http://www.genecodes.com

6
http://beast.bio.ed.ac.uk/Tracer

7
https://www.ncbi.nlm.nih.gov/sra/PRJNA626668

8
https://github.com/Bioflora/Loliinae_plastomes

9
https://github.com/Bioflora/Podophorus_plastome

## Supplementary Material

FIGURE S1 **(A)** Maximum likelihood full plastome cladogram (35 Loliinae taxa, *Podophorus* excluded) constructed with IQTREE showing the relationships among the studied Fernandezian and Loliinae grasses. *Oryza sativa* was used to root the trees. Numbers indicate branches with UltraFast Bootstrap supports (BS). **(B)** Maximum likelihood reduced plastome cladogram (36 Loliinae taxa, *Podophorus* included) constructed with IQTREE showing the relationships among the studied Fernandezian and Loliinae grasses. *Oryza sativa* was used to root the trees. Numbers indicate branches with UltraFast Bootstrap supports (BS). **(C)** Maximum likelihood nuclear rDNA cistron cladogram (35 Loliinae taxa, *Podophorus* excluded) constructed with IQTREE showing the relationships among the studied Fernandezian and Loliinae grasses. *Oryza sativa* was used to root the trees. Numbers indicate branches with UltraFast Bootstrap supports (BS). **(D)** Maximum likelihood nuclear ITS cladogram (36 Loliinae taxa, *Podophorus* included) constructed with IQTREE showing the relationships among the studied Fernandezian and Loliinae grasses. *Oryza sativa* was used to root the trees. Numbers indicate branches with UltraFast Bootstrap supports (BS).

FIGURE S2 **(A)** Maximum likelihood nuclear TLF tree (135 Loliinae taxa) constructed with IQTREE showing the relationships among the studied Fernandezian and Loliinae grasses. *Oryza sativa* was used to root the trees. Numbers indicate branches with UltraFast Bootstrap supports (BS) <100%; the remaining branches have 100% BS values. **(B)** Maximum likelihood plastid ITS tree (135 Loliinae taxa) constructed with IQTREE showing the relationships among the studied Fernandezian and Loliinae grasses. *Oryza sativa* was used to root the trees. Numbers indicate branches with UltraFast Bootstrap supports (BS) <100%; the remaining branches have 100% BS values. **(C)** Maximum likelihood combined ITS-TLF tree (135 Loliinae taxa) constructed with IQTREE showing the relationships among the studied Fernandezian and Loliinae grasses. *Oryza sativa* was used to root the trees. Numbers indicate branches with UltraFast Bootstrap supports (BS).

FIGURE S3 Fully expanded Bayesian maximum clade credibility dated chronogram of 135 Loliinae taxa constructed with BEAST2 using nuclear ITS and plastid TLF loci showing estimated nodal divergence times (medians, in Ma) and 95% highest posterior density (HPD) intervals (bars) above branches and Posterior Probability Support (PPT) values below branches. Stars indicate secondary nodal calibration priors (means ± SD, in Mya) for the crown nodes of the BOP, *Brachypodium* + core pooids, and fine-leaved Loliinae clades.

TABLE S1 List of taxa included in the phylogenetic study of the Fernandezian and other Loliinae grasses. Taxon, source, ploidy level, nuclear ITS, plastid *trn*TL and *trn*LF, plastome and rDNA cistron Genbank codes, average alignment insert size, total number of pair-end reads, number of plastome assembled reads, and number of rDNA cistron assembled reads are indicated for the corresponding samples.

TABLE S2 **(A)** Operational areas used in the stratified Loliinae DEC Lagrange analysis. **(B)** Dispersal rate matrices reflecting the palaeogeographic connectivity among the study areas in each historical scenario (time slices TSI, TSII, TSIII, TIV). **(C)** Operational areas used in the stratified American-Vulpia-Pampas DEC Lagrange analysis. **(D)** Dispersal rate matrices reflecting the palaeogeographic connectivity among the study areas in each historical scenario (time slices TSIII, TIV).
