# Corrigendum: Museomics Unveil the Phylogeny and Biogeography of the Neglected Juan Fernandez Archipelago *Megalachne* and *Podophorus* Endemic Grasses and Their Connection With Relict Pampean-Ventanian Fescues

**DOI:** 10.3389/fpls.2021.817266

**Published:** 2021-12-16

**Authors:** María Fernanda Moreno-Aguilar, Itziar Arnelas, Aminael Sánchez-Rodríguez, Juan Viruel, Pilar Catalán

**Affiliations:** ^1^Escuela Politécnica Superior de Huesca, Universidad de Zaragoza, Huesca, Spain; ^2^Departamento de Ciencias Biológicas, Universidad Técnica Particular de Loja, Loja, Ecuador; ^3^Royal Botanic Gardens, Kew, Richmond, United Kingdom; ^4^Grupo de Bioquímica, Biofísica y Biología Computacional (BIFI, UNIZAR), Unidad Asociada al CSIC, Zaragoza, Spain; ^5^Department of Botany, Institute of Biology, Tomsk State University, Tomsk, Russia

**Keywords:** ancestral range reconstruction, endemic Loliinae grasses, Fernandezian clade, genome skimming, phylogenomics, taxonomically neglected species

In the original article, there was a mistake in Table 1, Figures 2A, 2B, 3A, 3B and 5, Supplementary Figures 1A, 1B, 1C, 1D, 2A, 2B, 2C and 3, and Supplementary Tables 1 and 2A as published. The name and authorship of *Festuca fontqueri* St-Yves were misspelled as *Festuca fontqueriana* (St-Yves) Romo.

**Table 1:** List of taxa included in the phylogenomic study of the Fernandezian and other Loliinae grasses.

**Taxon**	**Source**	**Ploidy**	**No. reads**	**Genbank/Phytozome accession No**.	
				**Plastome**	**rDNA cistron**
*Festuca abyssinica*	Tanzania: Kilimanjaro	4x	12041	SAMN14647043	MT145276
*Festuca africana*	Uganda: Bwindi forest	10x	13549	SAMN14647044	MT145277
*Festuca amplissima*	Mexico: Barranca del Cobre	6x	12058	SAMN14647045	MT145278
*Festuca arundinacea* var. *letourneuxiana*	Morocco: Atlas Mountains	10x	16839	SAMN14647059	MT145292
*Festuca asplundii*	Ecuador: Saraguro	6x	25088	SAMN14647046	MT145279
*Festuca caldasii*	Ecuador: Las Chinchas –Tambara	?	9863	SAMN14647047	MT145280
*Festuca capillifolia*	España: Cazorla	2x	13430	SAMN14647048	MT145281
*Festuca chimborazensis*	Ecuador: Chimborazo-Cotopaxi	4x	10913	SAMN14647049	MT145282
*Festuca durandoi*	Portugal: Alto do Espinheiro	2x	12688	SAMN14647050	MT145283
*Festuca eskia*	Spain: Picos de Europa	2x	24041	SAMN14647051	MT145284
*Festuca fenas*	Spain: Madrid	4x	16112	SAMN14647052	MT145285
*Festuca fimbriata*	Argentina: Apóstoles	6x	15741	SAMN14647053	MT145286
*Festuca fontqueri*	Morocco: Rif, Outa-El-Kadir	2x	22187	SAMN14647054	MT145287
*Festuca gracillima*	Argentina: Tierra de Fuego	6x	13888	SAMN14647055	MT145288
*Festuca holubii*	Ecuador: Saraguro	?	10264	SAMN14647056	MT145289
*Festuca francoi*	Portugal: Azores	2x	17592	SAMN14647057	MT145290
*Festuca lasto*	Spain: Los Alcornocales	2x	21581	SAMN14647058	MT145291
*Festuca mairei*	Morocco: Atlas Mountains	4x	19134	SAMN14647060	MT145293
*Festuca molokaiensis*	USA: Molokai	?	12188	SAMN14647061	MT145294
*Festuca ovina*	Russia: Gatchinskii Raion	2x	11364	SAMN14647062	MT145295
*Festuca pampeana*	Argentina: Sierra Ventana	6x	14862	SAMN14647063	MT145296
*Festuca paniculata*	Spain: Puerto de los Castaños	2x	35808	SAMN14647064	MT145297
*Festuca parvigluma*	China: Baotianman	4x	15872	SAMN14647065	MT145298
*Festuca pratensis*	England: USDA/283306	2x	30021	SAMN14647066	MT145301
*Festuca procera*	Ecuador: Riobamba	4x	12189	SAMN14647067	MT145299
*Festuca pyrenaica*	Spain: Pyrenees, Tobacor	4x	40669	SAMN14647068	MT145300
*Festuca pyrogea*	Argentina: Tierra de fuego	?	16835	SAMN14647069	MT145302
*Festuca quadridentata*	Ecuador: Chimborazo	?	15091	SAMN14647070	MT145303
*Festuca spectabilis*	Bosnia-Hercegovina: Troglav	6x	12960	SAMN14647071	MT145304
*Festuca superba*	Argentina: Jujuy, Yala	8x	12193	SAMN14647072	MT145305
*Festuca triflora*	Morocco: Rif, Ketama	2x	24472	SAMN14647073	MT145306
*Megalachne berteroniana*	Chile: JuanFernandez, Masatierra	?	5288	SAMN14647074	MT145307
*Megalachne masafuerana*	Chile: JuanFernandez, Masafuera	?	6134	SAMN14647075	MT145308
*Podophorus bromoides*	Chile: JuanFernandez, Masatierra	?	6694	SAMN14668162	——
*Vulpia ciliata*	Spain: Mar de Ontígola	4x	11801	SAMN14647076	MT145309
*Vulpia sicula*	Italia: Sicilia, Madone	2x	11327	SAMN14647077	MT145310
Outgroups					
*Brachypodium distachyon*	Iraq: near Salakudin	2x	-	NC_011032.1	phytozome.jgi.doe.gov, Bd21 v.3.1
*Oryza sativa* subsp. *japonica*	cv. PA64S; cv. Nipponbare	2x	-	AY522331.1	AP008215

**Figure 2:**
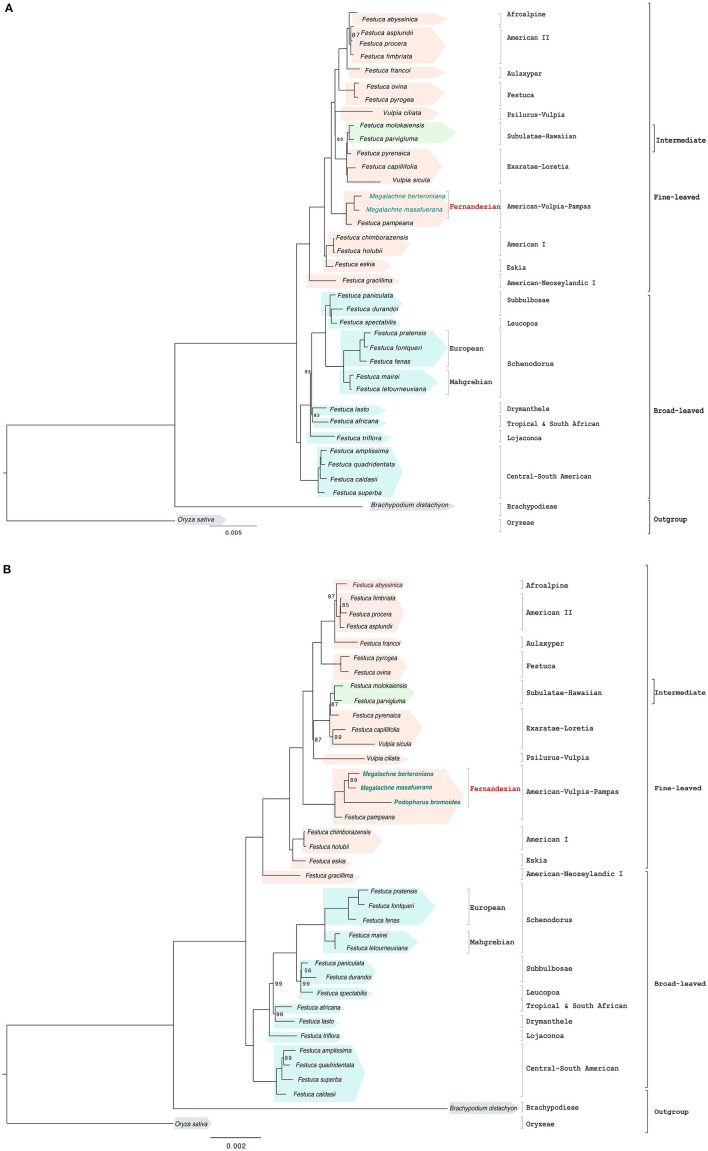
Maximum likelihood full plastome **(A)** and reduced plastome **(B)** trees constructed with IQTREE showing the relationships among the studied Fernandezian and Loliinae grasses. Oryza sativa was used to root the trees. Numbers indicate branches with UltraFast Bootstrap supports (BS) <100%; the remaining branches have 100% BS value.

**Figure 3:**
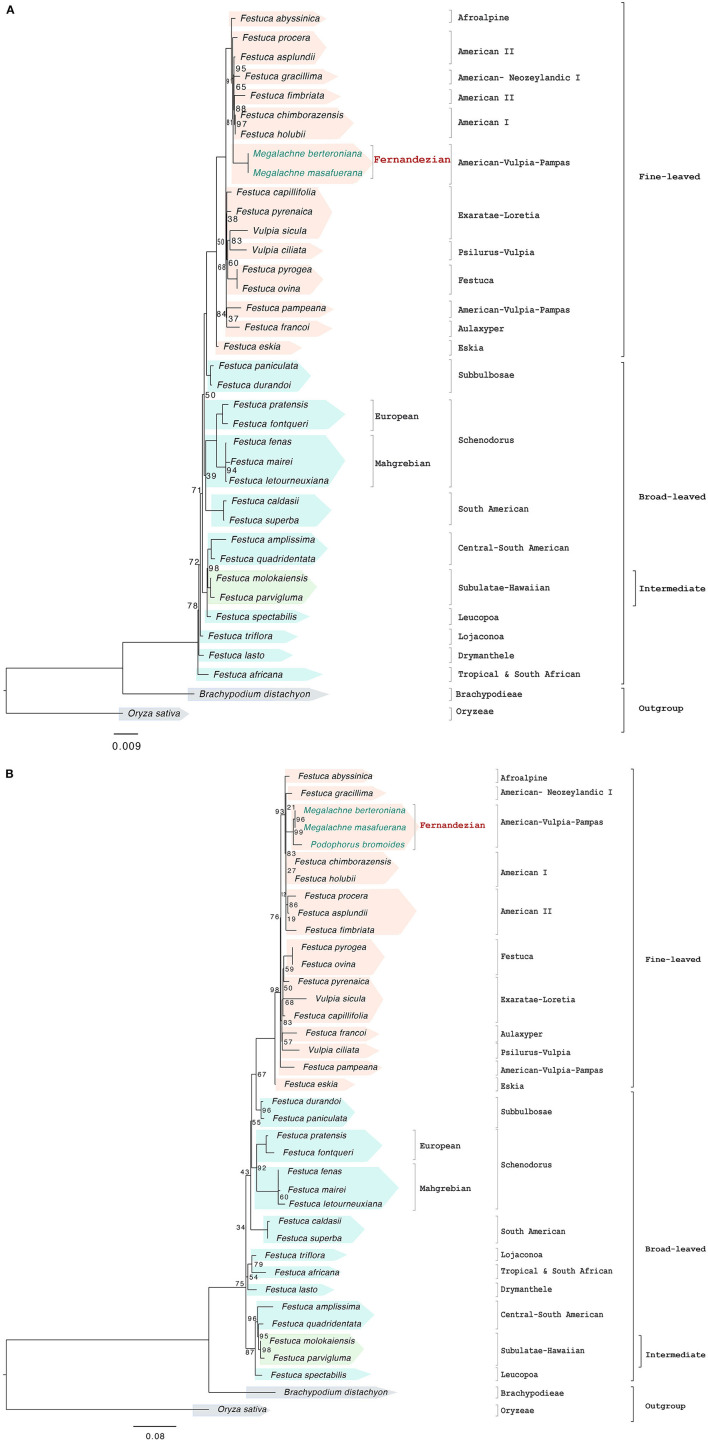
Maximum likelihood nuclear rDNA cistron **(A)** and ITS **(B)** trees constructed with IQTREE showing the relationships among the studied Fernandezian and Loliinae grasses. *Oryza sativa* was used to root the trees. Numbers indicate branches with UltraFast Bootstrap supports (BS) <100%; the remaining branches have 100% BS value.

**Figure 5:**
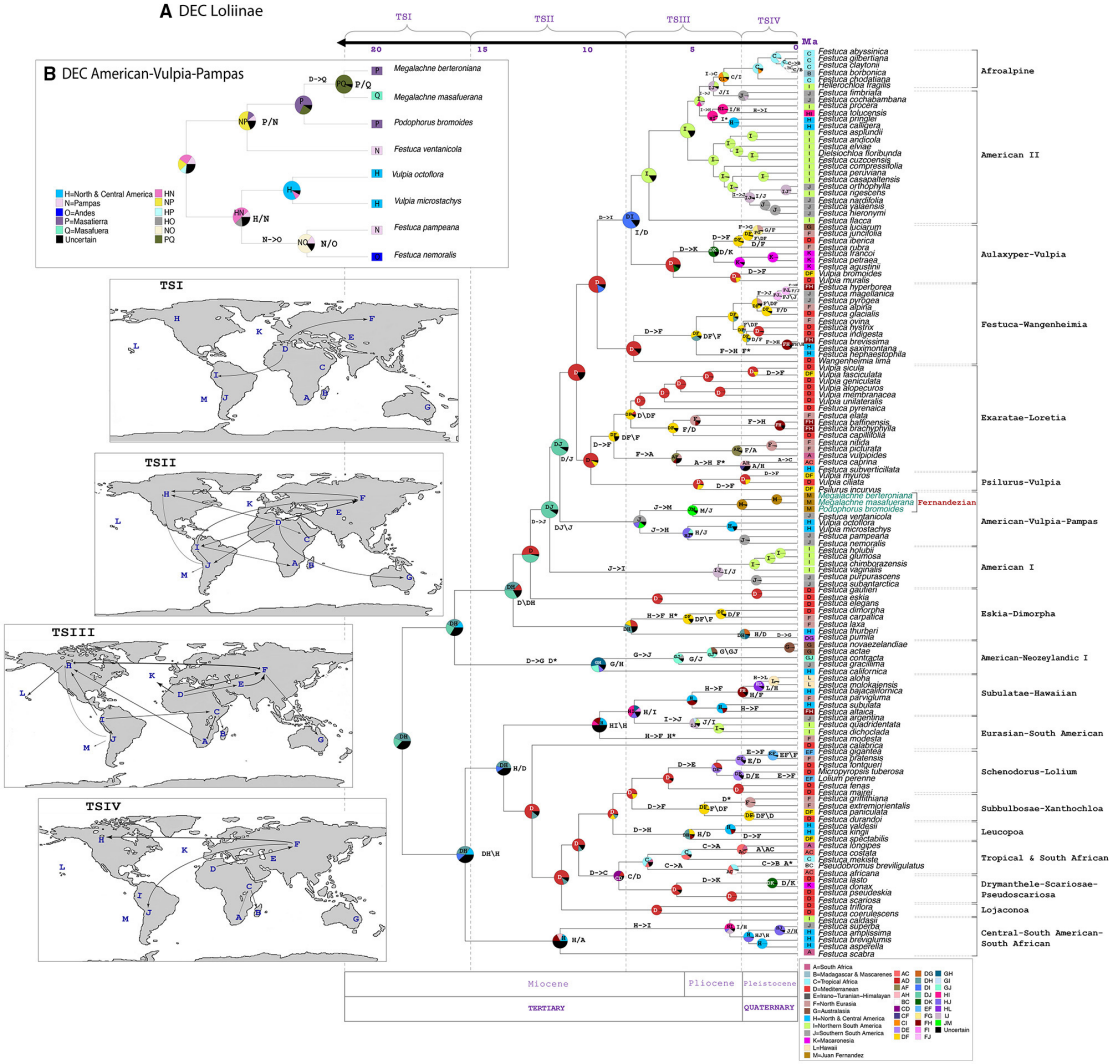
Estimated ancestral ranges and biogeographical events of the Fernandezian and other Loliinae grasses inferred from LAGRANGE under the stratified DEC models mapped on the BEAST2 maximum clade credibility tree with outgroups pruned from it. **(A)** Loliinae DEC model; **(B)** American-Vulpia-Pampas DEC model. The vertical dashed lines separate the four time slices (TSI-TSIV) used in the Lagrange analyses. The maps on the left represent the palaeogeographical configuration of the world in these four time periods and the arrows represent the dispersals between areas that reflect change in continental connectivity over time. The pie charts at the nodes indicate the relative probabilities for alternative ancestral ranges (with their color legends indicated at the respective inset charts). The inferred biogeographic events are indicated at the nodes (X/Y vicariance; X/Y peripheral isolation) and branches (X->Y dispersal; X^*^ extinction) of the tree. The Operational Areas assigned to the species are indicated to the right of the trees.

In addition, the GenBank accession codes of ITS, trnLF and trnTL DNA sequences of *Avena fatua, Oryza sativa, Poa alpina, Secale cereale* and *Triticum aestivum* were wrong in Supplementary Table 1.

The corrected Table 1, Figures 2A, 2B, 3A, 3B and 5 appear below and corrected Supplementary Figures 1A, 1B, 1C, 1D, 2A, 2B, 2C and 3, and Supplementary Tables 1 and 2A are published.

The authors apologize for this error and state that this does not change the scientific conclusions of the article in any way. The original article has been updated.

## Publisher's Note

All claims expressed in this article are solely those of the authors and do not necessarily represent those of their affiliated organizations, or those of the publisher, the editors and the reviewers. Any product that may be evaluated in this article, or claim that may be made by its manufacturer, is not guaranteed or endorsed by the publisher.

