# Supplementary material for: Museomics Unveil the Phylogeny and Biogeography of the Neglected Juan Fernandez Archipelago Megalachne and Podophorus Endemic Grasses and Their Connection With Relict Pampean-Ventanian Fescues
Source: Front Plant Sci. 2020 Jun 26;11:819. doi: 10.3389/fpls.2020.00819 (PMC7333454; doi:10.3389/fpls.2020.00819)
Supplement: FIGURE S1 — (A) Maximum likelihood full plastome cladogram (35 Loliinae taxa, Podophorus excluded) constructed with IQTREE showing the relationships among the studied Fernandezian and Loliinae grasses. Oryza sativa was used to root the trees. Numbers indicate branches with UltraFast Bootstrap supports (BS). (B) Maximum likelihood reduced plastome cladogram (36 Loliinae taxa, Podophorus included) constructed with IQTREE showing the relationships among the studied Fernandezian and Loliinae grasses. Oryza sativa was used to root the trees. Numbers indicate branches with UltraFast Bootstrap supports (BS). (C) Maximum likelihood nuclear rDNA cistron cladogram (35 Loliinae taxa, Podophorus excluded) constructed with IQTREE showing the relationships among the studied Fernandezian and Loliinae grasses. Oryza sativa was used to root the trees. Numbers indicate branches with UltraFast Bootstrap supports (BS). (D) Maximum likelihood nuclear ITS cladogram (36 Loliinae taxa, Podophorus included) constructed with IQTREE showing the relationships among the studied Fernandezian and Loliinae grasses. Oryza sativa was used to root the trees. Numbers indicate branches with UltraFast Bootstrap supports (BS). [file Data_Sheet_1.pdf]

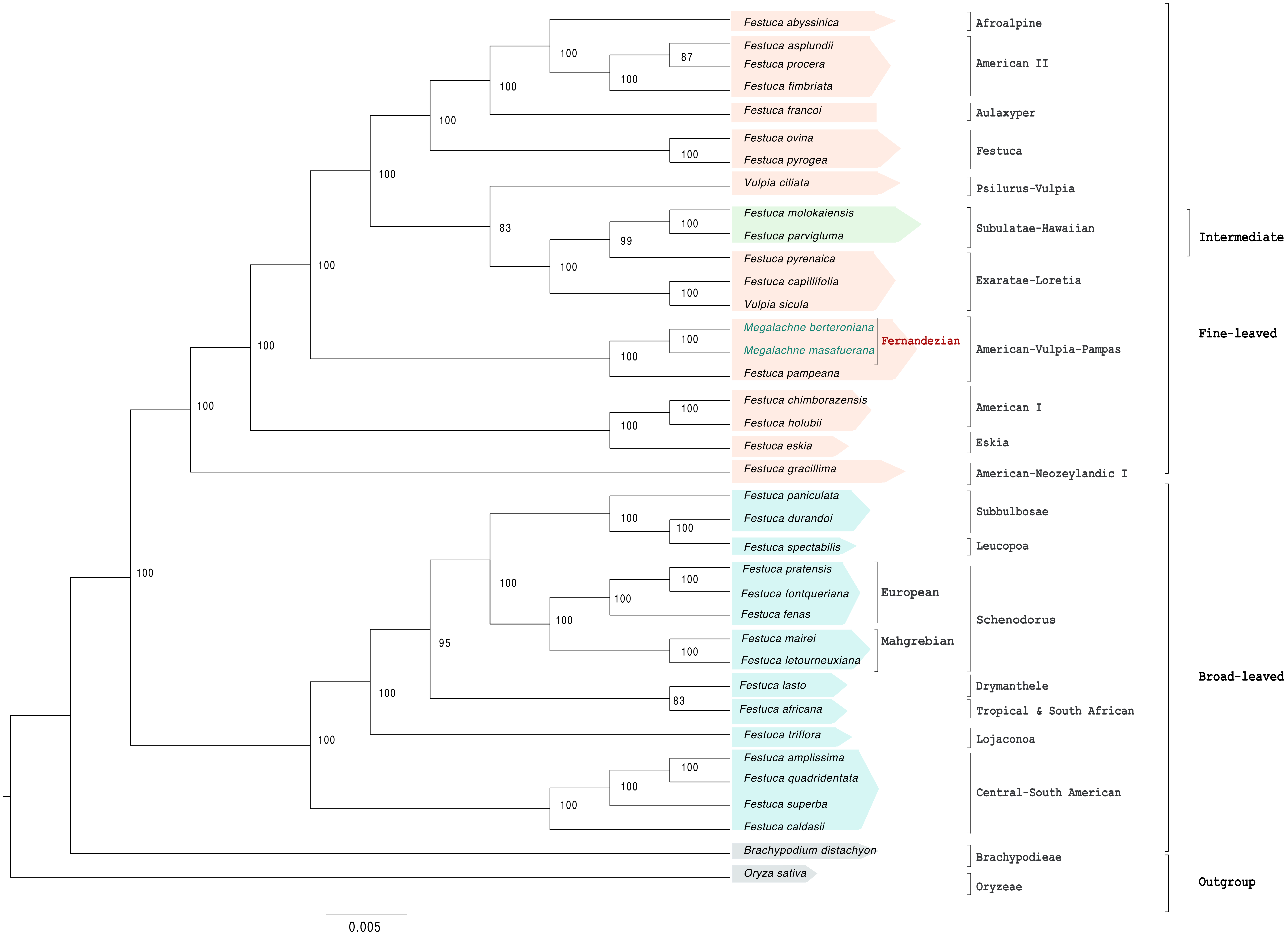

Suppl. Fig. 1A Maximum likelihood full plastome cladogram (35 Loliinae taxa, *Podophorus* excluded) constructed with IQTREE showing the relationships among the studied Fernandezian and Loliinae grasses. *Oryza sativa* was used to root the trees. Numbers indicate branches with UltraFast Bootstrap supports (BS).

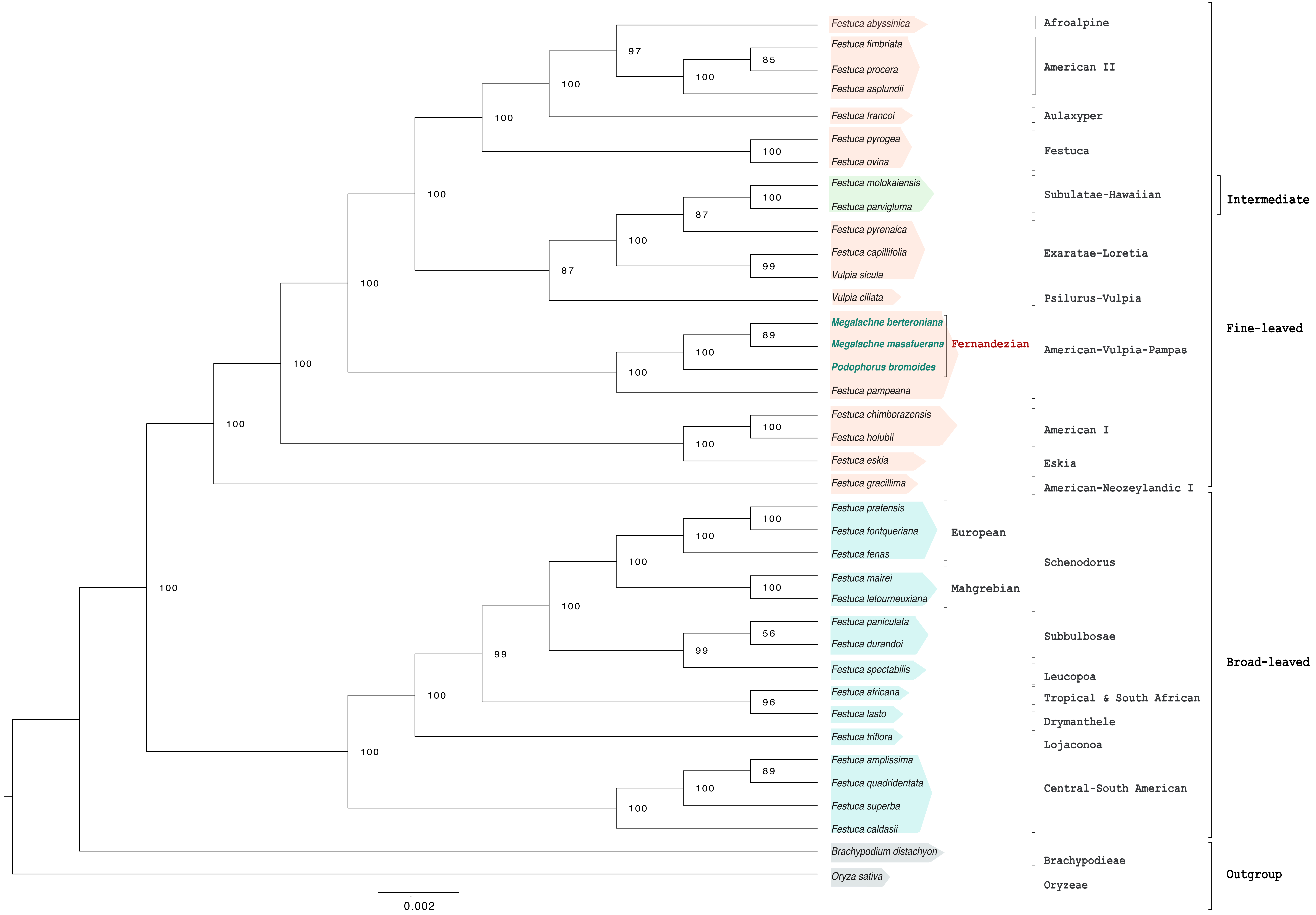

Suppl. Fig. 1B. Maximum likelihood reduced plastome cladogram (36 Loliinae taxa, *Podophorus* included) constructed with IQTREE showing the relationships among the studied Fernandezian and Loliinae grasses. *Oryza sativa* was used to root the trees. Numbers indicate branches with UltraFast Bootstrap supports (BS).

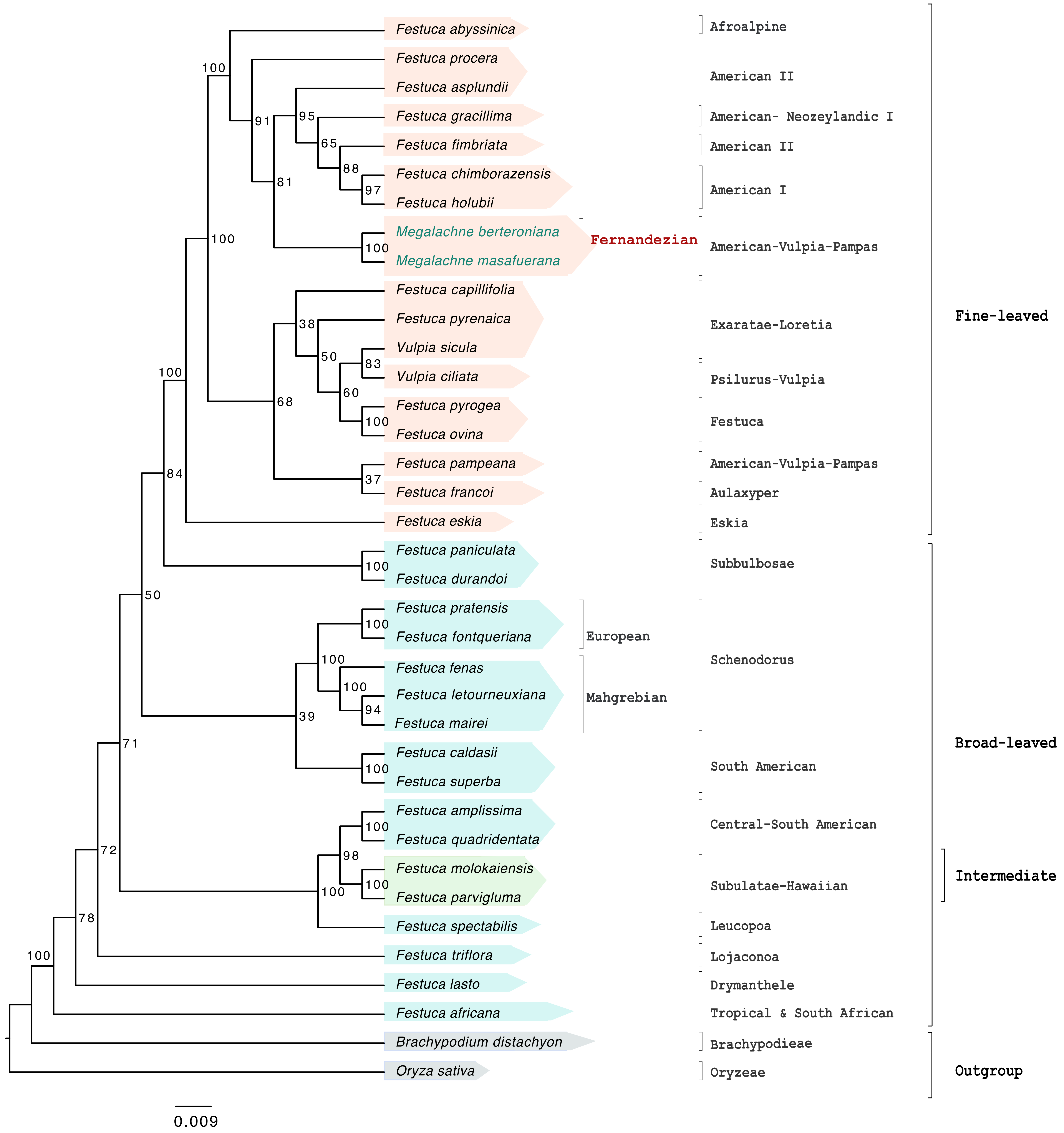

Suppl. Fig. 1C. Maximum likelihood nuclear rDNA cistron cladogram (35 Loliinae taxa, *Podophorus* excluded) constructed with IQTREE showing the relationships among the studied Fernandezian and Loliinae grasses. *Oryza sativa* was used to root the trees. Numbers indicate branches with UltraFast Bootstrap supports (BS).

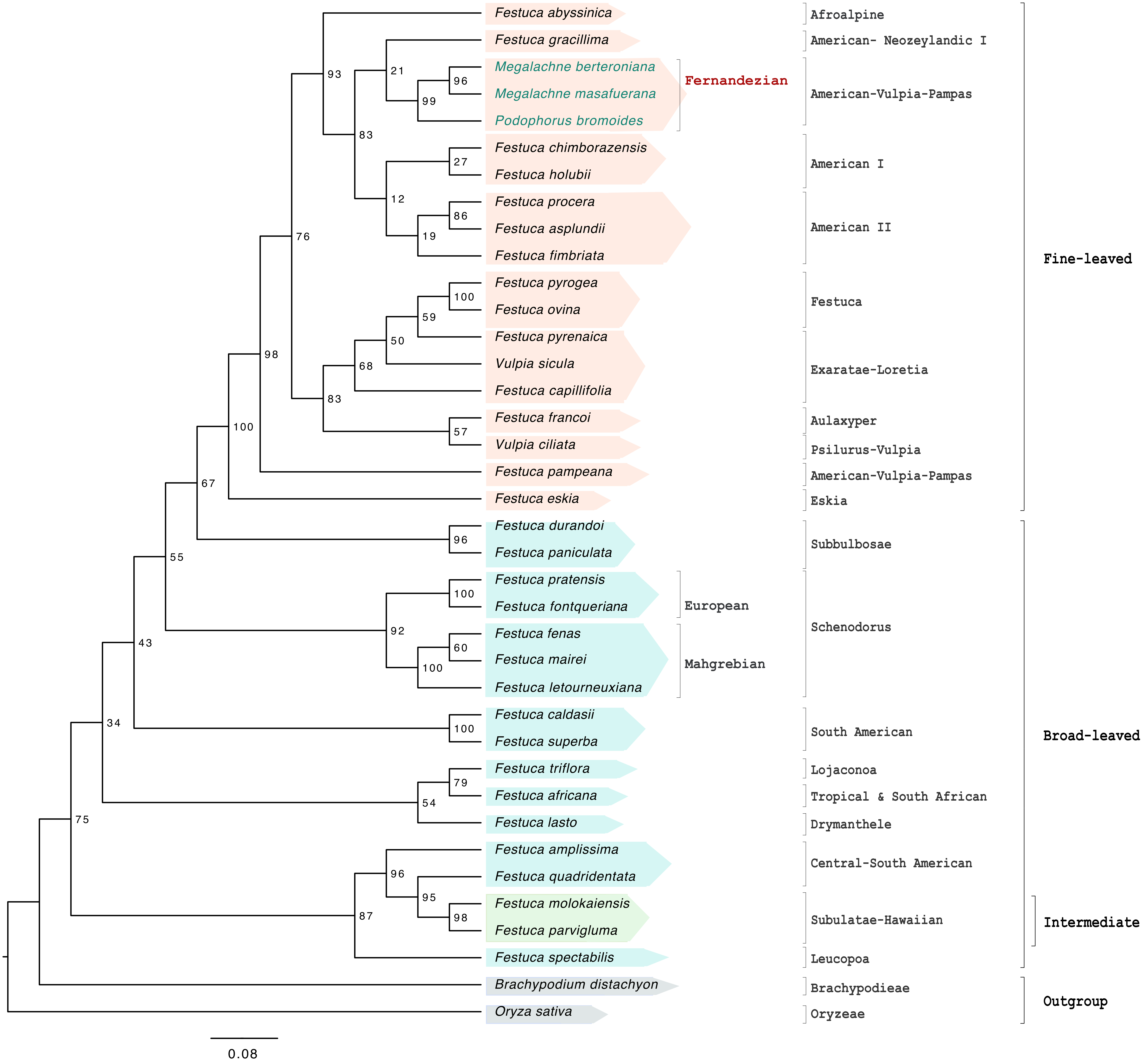

Suppl. Fig. 1D. Maximum likelihood nuclear ITS cladogram (36 Loliinae taxa, *Podophorus* included) constructed with IQTREE showing the relationships among the studied Fernandezian and Loliinae grasses.
Oryza sativa was used to root the trees. Numbers indicate branches with UltraFast Bootstrap supports (BS).

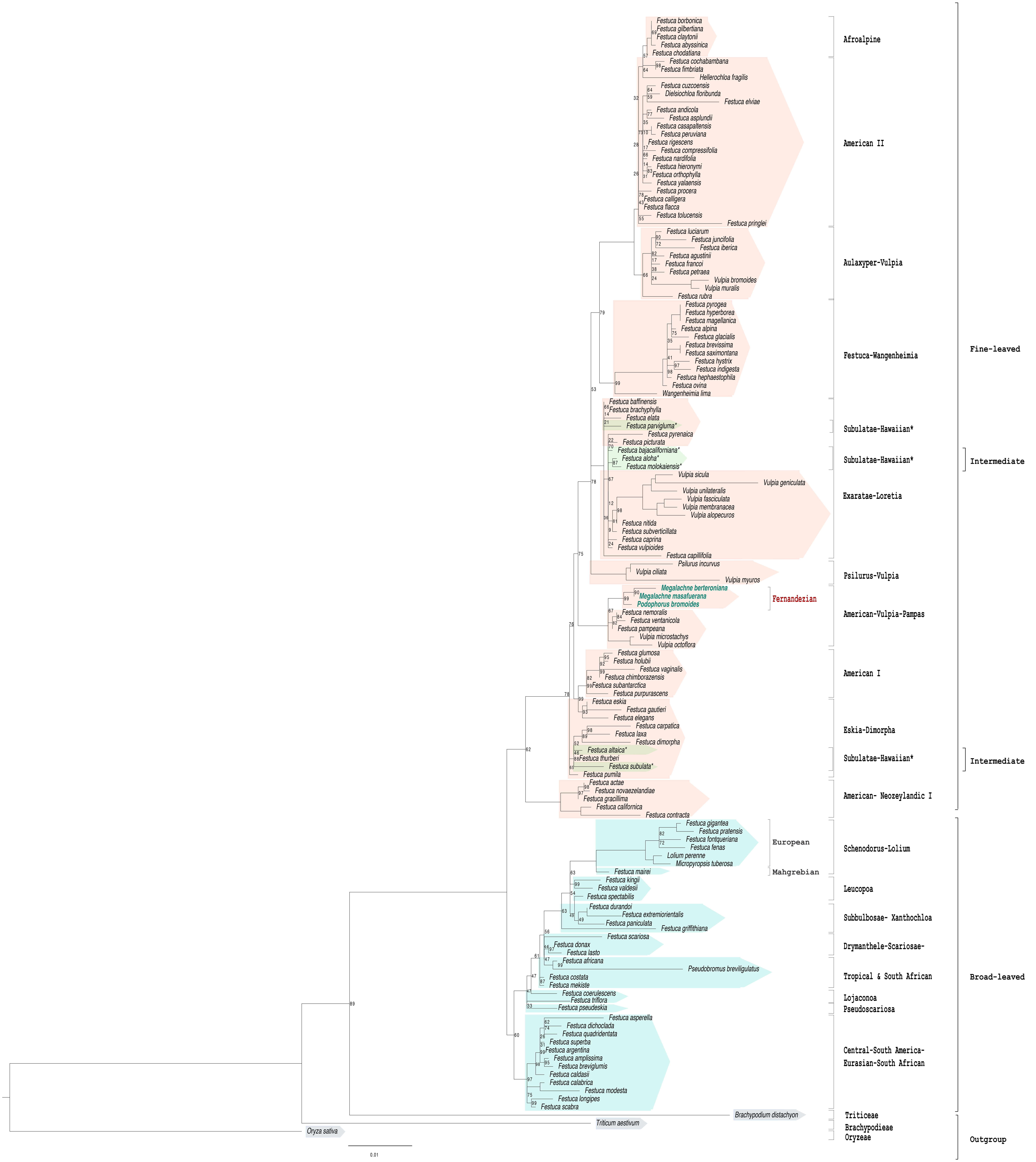

Suppl. Fig. 2A. Maximum likelihood nuclear TLF tree (135 Loliinae taxa) constructed with IQTREE showing the relationships among the studied Fernandezian and Loliinae grasses. *Oryza sativa* was used to root the trees. Numbers indicate branches with UltraFast Bootstrap supports (BS) <100%; the remaining branches have 100% BS values.

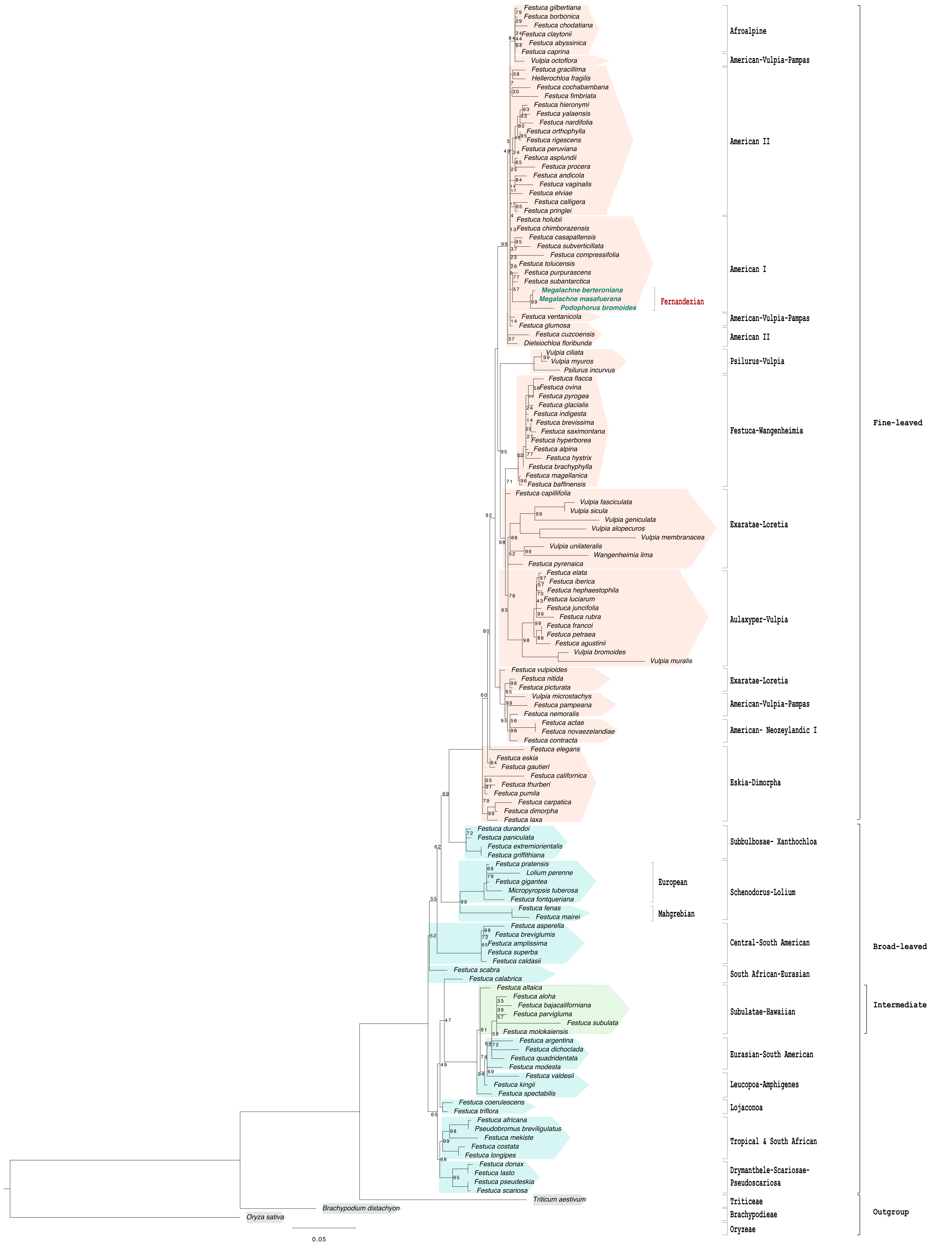

Suppl. Fig. 2B. Maximum likelihood plastid ITS tree (135 Loliinae taxa) constructed with IQTREE showing the relationships among the studied Fernandezian and Loliinae grasses. *Oryza sativa* was used to root the trees. Numbers indicate branches with UltraFast Bootstrap supports (BS) <100%; the remaining branches have 100% BS values.

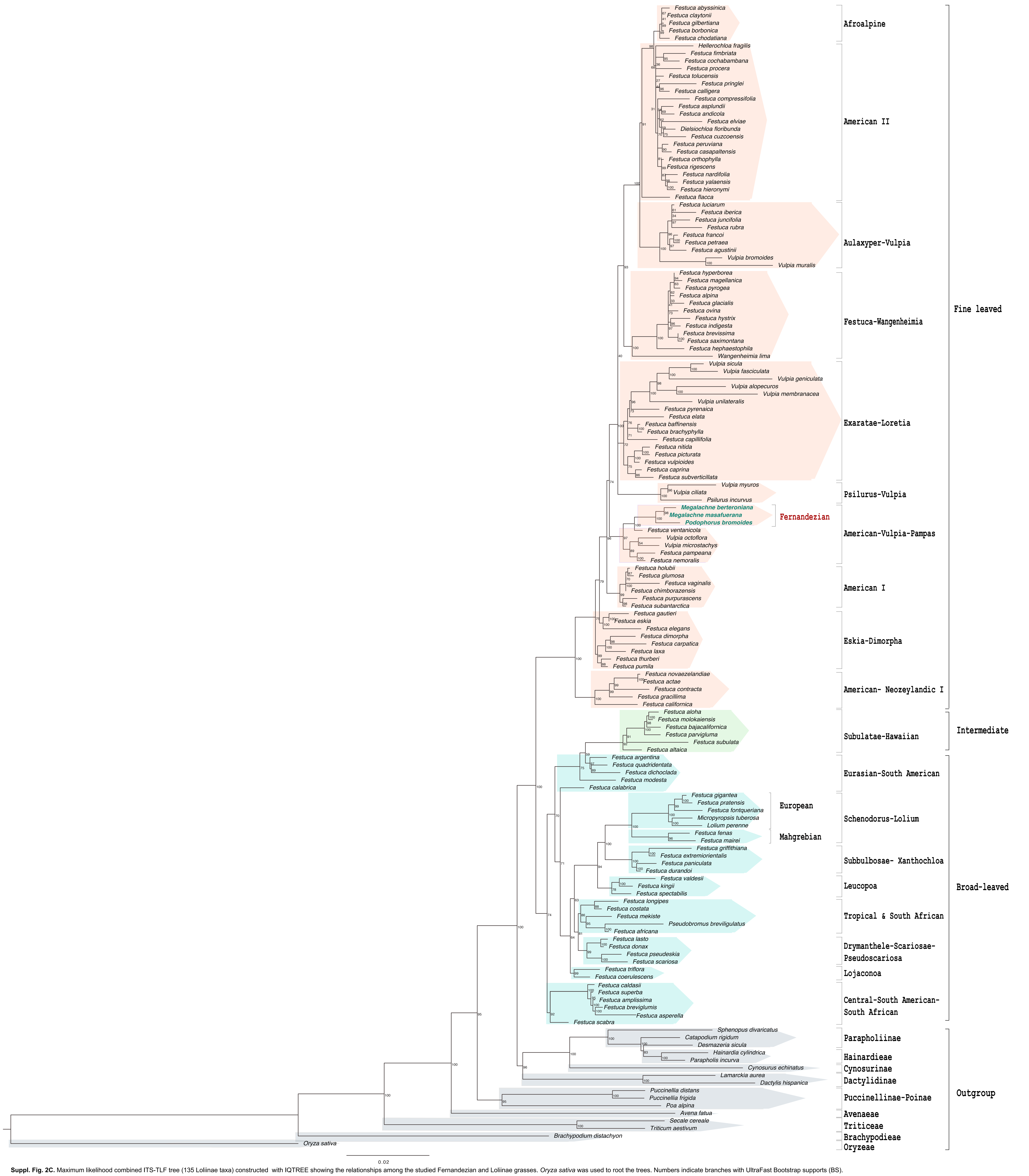

Suppl. Fig. 2C. Maximum likelihood combined ITS-TLF tree (135 Loliinae taxa) constructed with IQTREE showing the relationships among the studied Fernandezian and Loliinae grasses. *Oryza sativa* was used to root the trees. Numbers indicate branches with UltraFast Bootstrap supports (BS).

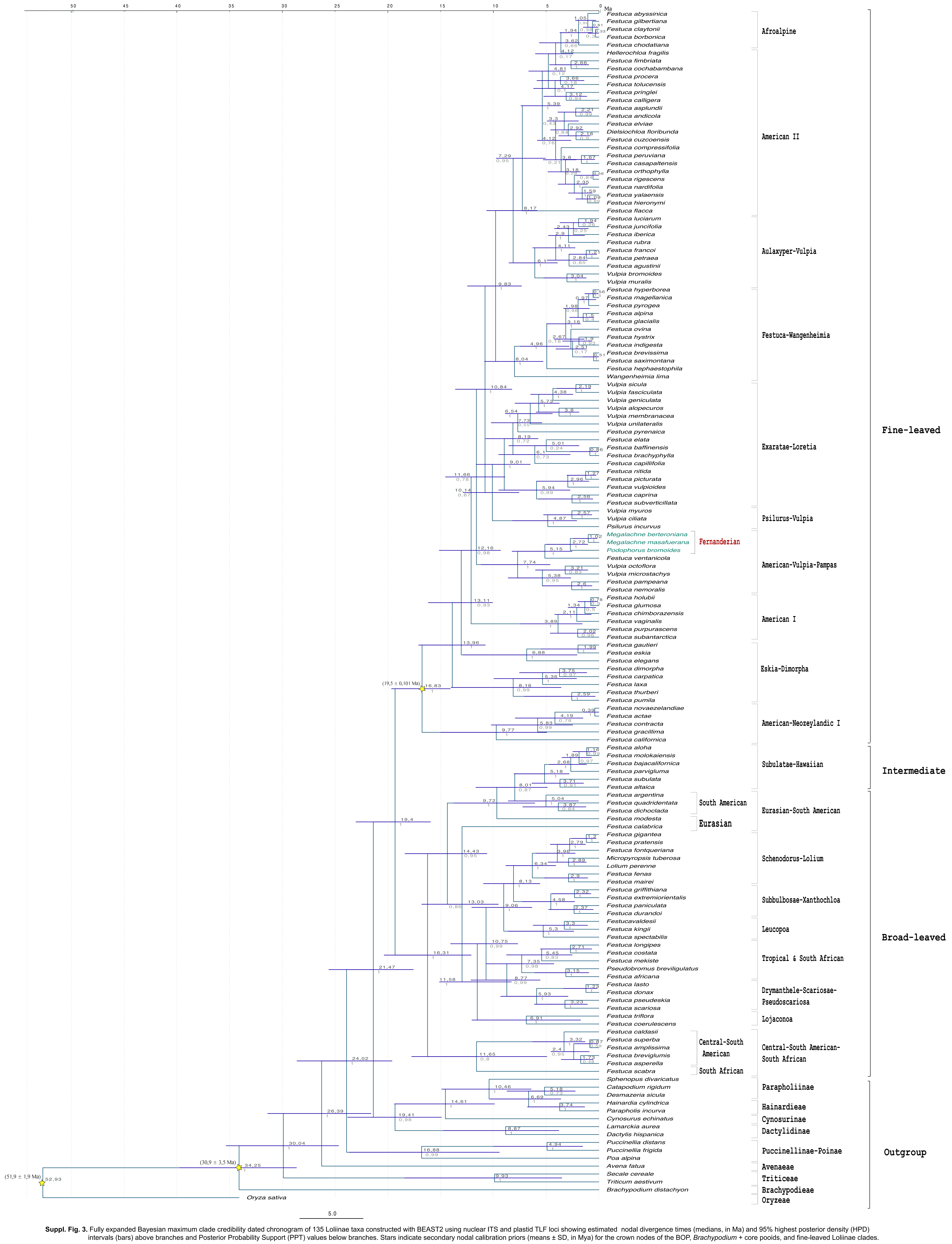

Suppl. Fig. 3. Fully expanded Bayesian maximum clade credibility dated chronogram of 135 Loliinae taxa constructed with BEAST2 using nuclear ITS and plastid TLF loci showing estimated nodal divergence times (medians, in Ma) and 95% highest posterior density (HPD) intervals (bars) above branches and Posterior Probability Support (PPT) values below branches. Stars indicate secondary nodal calibration priors (means ± SD, in Mya) for the crown nodes of the BOP, *Brachypodium* + core pooids, and fine-leaved Loliinae clades.
